# Impact of stimulation among non-crying neonates with intact cord versus clamped cord on birth outcomes: observation study

**DOI:** 10.1136/bmjpo-2021-001207

**Published:** 2021-01-15

**Authors:** Ashish KC, Shyam Sundar Budhathoki, Jeevan Thapa, Susan Niermeyer, Rejina Gurung, Nalini Singhal, Nepal Neonatal Network, Omkar Basnet

**Affiliations:** 1 Department of Women’s and Children’s Health, Uppsala University, Uppsala, Sweden; 2 Department of Primary Care and Public Health, Imperial College London, London, UK; 3 Research Divison, Golden Community, Jawgal, Lalitpur, Nepal; 4 Department of Community Health Sciences, Patan Academy of Health Sciences, Kathmandu, Nepal; 5 Pediatrics, University of Colorado Denver School of Medicine, Aurora, Colorado, USA; 6 Pediatrics, University of Calgary, Calgary, Alberta, Canada

**Keywords:** resuscitation, epidemiology, neonatology

## Abstract

**Background:**

Stimulation of non-crying neonates after birth can help transition to spontaneous breathing. In this study, we aim to assess the impact of intact versus clamped umbilical cord on spontaneous breathing after stimulation of non-crying neonates.

**Methods:**

This is an observational study among non-crying neonates (n=3073) born in hospitals of Nepal. Non-crying neonates born vaginally at gestational age ≥34 weeks were observed for their response to stimulation with the cord intact or clamped. Obstetric characteristics of the neonates were analysed. Association of spontaneous breathing with cord management was assessed using logistic regression.

**Results:**

Among non-crying neonates, 2563 received stimulation. Of these, a higher proportion of the neonates were breathing in the group with cord intact as compared with the group cord clamped (81.1% vs 68.9%, p<0.0001). The use of bag-and-mask ventilation was lower among those who were stimulated with the cord intact than those who were stimulated with cord clamped (18.0% vs 32.4%, p<0.0001). The proportion of neonates with Apgar Score ≤3 at 1 min was lower with the cord intact than with cord clamped (7.6% vs 11.5%, p=0.001). In multivariate analysis, neonates with intact cord had 84% increased odds of spontaneous breathing (adjusted OR, 1.84; 95% CI: 1.48 to 2.29) compared with those with cord clamped.

**Conclusions:**

Stimulation of non-crying neonates with intact cord was associated with more spontaneous breathing than among infants who were stimulated with cord clamped. Intact cord stimulation may help establish spontaneous breathing in apnoeic neonates, but residual confounding variables may be contributing to the findings. This study provides evidence for further controlled research to evaluate the effect of initial steps of resuscitation with cord intact.

**What is known about the subject?**

- Stimulation is one of the key steps to neonatal resuscitation.
- Breathing prior to umbilical cord clamping has been shown to result in smoother cardiovascular transition at birth in experimental studies.figure

**What this study adds?**

- Intact cord stimulation to breathe with the cord intact may help deliver intervention more quickly than stimulation after cord clamping and may also avoid the reflex bradycardia that can occur when the cord is clamped before the lungs are aerated. Further research is needed to define if the results are confounded by factors such as provider experience or the baby’s tone and colour influencing management decisions.

## Introduction

Globally, among the 140 million neonates who are born every year, 10–15 million do not cry or breathe at birth.^1^ These neonates may require resuscitation to transition from the intrauterine to the extrauterine environment.^2^ Neonates who do not receive timely and adequate resuscitation may die or suffer brain injuries and long-term disability.^3^ Every year, an estimated 1 million neonates die due to intrapartum-related complications or ‘birth asphyxia’,^4^ 2 million experience hypoxic-ischaemic encephalopathy and 1.2 million go on to show developmental delay.^3^ Most of these mortalities and morbidities occur in low-income and middle-income countries.

Transitioning to an extrauterine environment depends on two major physiological events, the commencement of breathing and the transition of blood flow from the umbilical to the pulmonary circulation.^2 5^ The trigger of breathing at birth results in clearance of the liquid in the trachea and airways, aeration of the lungs, reduction in pulmonary vascular resistance and increase in pulmonary blood flow.^6 7^ This increase in pulmonary blood flow supplies left ventricular preload previously dependent on flow across the patent foramen ovale from the right atrium and the umbilical vein.^2^ Any intrapartum insult due to placental insufficiency (hypertension, infection or haemorrhage) can delay the spontaneous cardiopulmonary transition and may be compounded by umbilical cord clamping.^8 9^ As a result, blood flows continuously through the ductus arteriosus, bypassing the pulmonary circulation into the systemic circulation as right-to-left shunting.^6^ Resuscitation with cord intact may provide a smoother cardiopulmonary transition at birth.

The International Liaison Committee on Resuscitation (ILCOR) provides guidance on neonatal resuscitation.^10^ The ILCOR 2020 recommends that a neonate who is not crying or breathing with poor tone and heart rate less than 100 beats per minute should receive stimulation and clearing of airways (as needed) with intact cord.^10^ There is very little robust evidence on the impact of stimulation in neonates receiving resuscitation with the cord intact. The WHO recommends if there is experience in providing ventilation without cutting the umbilical cord, ventilation can be initiated before cord-cutting.^11^ WHO identifies resuscitation with the cord intact as a key research area to generate a stronger evidence base for care.^11^

Studies from low-income and middle-income countries have reported that neonates who are not crying or breathing at birth are immediately taken to a neonatal resuscitation area for stimulation, clearing of airways and positive-pressure ventilation.^12 13^ Despite educational programmes such as Helping Babies Breathe (HBB) from the American Academy of Pediatrics, which advocates stimulation to breathe before clamping the cord, health workers often clamp the cord before initiating resuscitation.^14^

This study aims to evaluate the impact of stimulation among neonates with the cord intact versus those with cord clamped on breathing and birth outcome.

## Methods

### Study design

This was a nested observational study within two quality improvement studies SUSTAIN^15^ and REFINE^16^ done in nine hospitals of Nepal.

### Study sites

This study was conducted among nine hospitals in Nepal. The hospitals are tertiary care hospitals providing referral obstetric services through comprehensive and emergency obstetric and neonatal care services. These hospitals were chosen for quality improvement studies to implement a safer birth bundle. Health workers were trained on HBB 2nd edition to resuscitate neonates with intact cord when possible as part of the safer birth bundle. The cord management was at the discretion of the individual health worker.

### Study dates

The study was conducted between 1 June 2019 and 2 May 2020.

### Participants

Women in labour with gestational age ≥34 weeks and confirmed fetal heart rate during admission were approached for consent. Women who were admitted for vaginal birth were approached for consent. For this study, neonates who were crying at birth were not eligible for analysis. Non-crying neonates who received additional tactile stimulation constituted the study population. Babies who cried immediately after birth and those with birth defects were excluded from the analysis. Neonates who did not cry and were not stimulated also were excluded from the study.

### Sample size

All non-crying neonates who received stimulation were considered for analysis.

### Variables

The variables considered were defined as cord status: intact and clamped; parity: no previous birth, one previous birth and two or more previous births; obstetrical complication during admission: includes pre-eclampsia, eclampsia, premature rupture of membranes, preterm labour, polyhydramnios, oligohydramnios, breech/transverse lie, decreased fetal movements, antepartum haemorrhage, chorioamnionitis, cord prolapse, meconium-stained amniotic fluid and maternal medical complications such as diabetes mellitus and pre-existing cardiovascular and renal disease; induction of labour, augmentation labour; mode of delivery: instrumental (for forceps, vacuum application) or spontaneous; preterm birth (age of gestation less than 37 weeks); low birth weight (weight of baby less than 2500 g); immediate thorough drying after birth and breathing after stimulation (spontaneous breathing requiring no positive-pressure ventilation). Stimulation was defined as additional rubbing of the back of non-crying neonates after drying. Stillbirth was defined as no heart rate or breathing at any time after birth.

### Data collection and management

The data collection was done through the existing data collection systems of the two studies REFINE and SUSTAIN. Observational data were collected by independent clinical researchers using a tablet-based application (online supplemental file 1). Observation was done on immediate newborn care, status of crying and when drying, clearing of the airway, stimulation and cord clamping were performed. Researchers also extracted information from patient notes on obstetric complications, progress of labour, mode of delivery, sex and weight of neonate. The data were then extracted into SPSS software for cleaning, coding and analysis.

10.1136/bmjpo-2021-001207.supp1 Supplementary data

### Data analysis

The non-crying babies included in the study were categorised as those who were stimulated with intact cord and those who were stimulated after cord clamping. Spontaneous breathing after stimulation was observed. Obstetrical and neonatal characteristics were compared between the two groups.

Multivariable analysis using logistic regression was done to assess the association of cord status during stimulation on breathing by adjusting for the variables which had a significance level difference of p<0.01. The variables which were significantly different by cord status during stimulation were complications during admission, mode of delivery and immediate drying.

### Patients and public involvement

The main study aimed to improve the provision and experience of care of patients (mother and newborn) during childbirth. Information was provided to patients on their involvement in the main study. Results from this study will be disseminated to healthcare providers, paediatric associations and global neonatal guideline development organisations.

## Results

**Figure 1:**
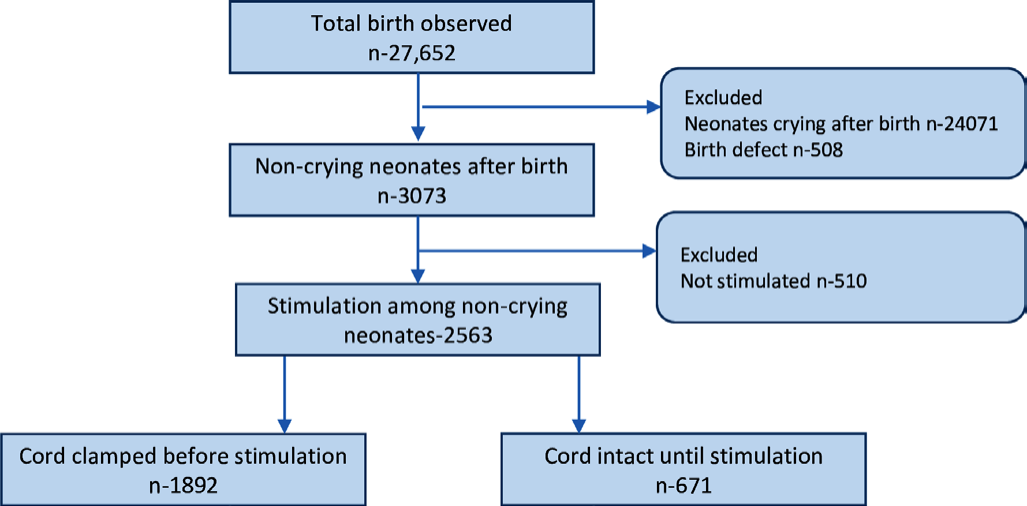
Study flow figure.

During the study period, 41 621 women delivered in the hospitals and 34 652 women consented to the study. Among the consented women, 27 652 births were observed in the study. Among the total births observed, 24 071 babies were excluded as they cried immediately after birth and 508 births were further excluded due to birth defects; 3073 non-crying neonates were included in the analysis. Among the non-crying neonates, 2563 were stimulated. Of the neonates stimulated, 671 (26.2%) of them had their cord intact, while 1892 (73.8%) had their cord clamped (figure 1).

We compared the obstetric and neonatal characteristics of neonates who had cord intact versus cord clamped before stimulation. Obstetric complications during admission were lower among neonates who had cord intact than those who had the cord clamped (8.0% vs 11.2%, p=0.022).

Neonates who had cord intact had lower proportion of breech or transverse lie during labour than those who had the cord clamped (1.3% vs 3.5%, p=0.003). Among neonates with cord intact, a lower proportion had meconium-stained amniotic fluid during labour as compared with those with the cord clamped (22.2% vs 28.4%, p=0.002). Induction of labour was lower among neonates with cord intact than clamped (18.5% vs 25.5%, p<0.0001). The proportion of neonates born through instrumental delivery was lower among those with cord intact clamped (8.4% vs 12.3%, p=0.005) (table 1).

**Table 1:** Obstetric and neonatal characteristics among non-crying neonates with intact cord versus cord clamped during stimulation

	Intact cord during stimulation, n=671	Cord clamped before stimulation, n=1892	P value
Median time to cord clamping (IQR), s	58.0 (34, 71)	25 (18, 31)	
Parity			
Nullipara, 1430	376 (56.5%)	1054 (56.0%)	0.892
Primipara, 714	180 (27.0%)	534 (28.4%)	0.515
Multipara, 403	110 (16.5%)	293 (15.6%)	0.579
Complication during admission			
No, 2292	617 (92.0%)	1675 (88.8%)	
Yes, 266	54 (8.0%)	212 (11.2%)	0.022
Antepartum haemorrhage, 5	5 (0.3%)	0 (0.0%)	0.335
Decreased fetal movements, 21	14 (0.7%)	7 (1.0%)	0.459
Pre-eclampsia and eclampsia, 49	34 (1.8%)	15 (2.24%)	0.291
Breech or transverse lie, 75	66 (3.5%)	9 (1.3%)	0.003
Prolapsed cord, 5	4 (0.2%)	1 (0.1%)	0.998
Chorioamnionitis, 18	12 (0.6%)	6 (0.9%)	0.59
Premature rupture of membrane, 43	38 (2.0%)	5 (0.7%)	0.034
Preterm labour, 87	68 (3.6%)	19 (2.8%)	0.387
Oligohydramnios, 28	18 (1.0%)	10 (1.5%)	0.279
Medical complication*, 79	50 (2.6%)	29 (4.3%)	0.037
Meconium-stained fluid			
No, 1876	1354 (71.6%)	522 (77.8%)	
Yes, 687	538 (28.4%)	149 (22.2%)	0.002
Induction of labour			
No, 1957	547 (81.5%)	1410 (74.5%)	
Yes, 606	124 (18.5%)	482 (25.5%)	<0.0001
Augmentation of labour			
No, 1600	445 (66.3%)	1155 (61.0%)	
Yes, 963	226 (33.7%)	737 (39.0%)	0.016
Mode of delivery			
Spontaneous, 2260	611 (91.6%)	1649 (87.7%)	
instrumental, 288	56 (8.4%)	232 (12.3%)	0.005
Gestational age in weeks (SD)	38.4±2.2	38.5±2.3	
Preterm (<37 weeks)			
No, 2272	594 (88.5%)	1678 (88.7%)	
Yes, 291	77 (11.5%)	214 (11.3%)	0.944
Birth weight in weeks (SD)	2784.3±543.7	2807.1±556.6	
Low birth weight (<2500 g)			
No, 2083	537 (82.3%)	1546 (82.3%)	
Yes, 463	131 (19.6%)	332 (17.7%)	0.268
Immediate drying			
No, 159	35 (5.2%)	124 (6.6%)	
Yes, 2404	636 (94.8%)	1892 (93.4%)	0.227

The clinical outcome differed among neonates stimulated with the cord intact versus with those cord clamped. Among neonates stimulated with the cord intact, 81.1% breathed, while 68.9% breathed after stimulation with cord clamped (p value<0.0001). The use of bag-and-mask ventilation was lower among those who were stimulated with the cord intact than those who were stimulated with cord clamped (18.0% vs 32.4%, p<0.0001). The proportion of neonates with Apgar Score 3 or less at 1 min was lower with the cord intact than clamped (7.6% vs 11.5% p=0.001). The proportion of neonates with Apgar Score 6 or less at 5 min was lower with the cord intact than clamped (7.9% vs 12. 7%, p<0.0001). The proportion of mortality was not significantly different between those with cord intact and clamped (0.7 vs 1.6%, p=0.123) (table 2).

**Table 2:** Clinical outcome of non-crying neonates who were stimulated with cord intact versus cord clamped

	Intact cord during stimulation, n=671 (26.2%)	Cord clamped before stimulation, n=1892 (73.8%)	P value
Spontaneous breathing			
Yes, 1847	544 (81.1%)	1303 (68.9%)	
No, 716	127 (18.9%)	589 (31.1%)	<0.0001
Bag-and-mask ventilation			
No, 1829	550 (82.0%)	1279 (67.6%)	
Yes, 734	121 (18.0%)	613 (32.4%)	<0.0001
Apgar Score at 1 min			
4 or more, 2214	598 (92.4%)	1616 (88.3%)	
3 or less, 264	49 (7.6%)	215 (11.5%)	0.001
Apgar Score at 5 min			
7 or more, 2196	596 (92.1%)	1597 (87.3%)	
6 or less, 284	51 (7.9%)	233 (12.7%)	<0.0001
Birth outcome			
Live born, 2513	654 (97.5%)	1859 (98.3%)	
Stillborn, 50	17 (2.5%)	33 (1.7%)	0.198
Mortality			
No, 2528	666 (99.3%)	1862 (98.4%)	
Yes, 35	5 (0.7%)	30 (1.6%)	0.123

We compared the obstetric and neonatal characteristics of non-crying neonates who breathed after stimulation. Overall, 58.3% of neonates with a maternal complication during admission breathed after stimulation, while 73.8% of neonates without maternal complication breathed after stimulation (p value<0.0001). Overall, 66.3% of neonates born through instrumental delivery breathed after stimulation, while 72.9% of neonates who were born through spontaneous delivery breathed after stimulation (p value=0.021). Among neonates who were not immediately dried after birth, 62.8% breathed after stimulation, while 72.7% of neonates who were immediately dried after birth breathed after additional stimulation (p value=0.01) (table 3).

**Table 3:** The obstetric and neonatal characteristics of non-crying neonates who responded to stimulation

	Breathing after stimulation (n=1847)	Not breathing after stimulation (n=716)	P value
Complication during admission			
No, 2292	1691(73.8%)	601(26.2%)	
Yes, 266	155(58.3%)	111(41.7%)	<0.0001
Induction of labour			
No, 1957	1406(71.8%)	551(28.2%)	
Yes, 606	441(72.8%)	165(27.2%)	0.679
Augmentation of labour			
No, 1600	1159(72.4%)	441(27.6%)	
Yes, 963	688(71.4%)	275(28.6%)	0.586
Mode of delivery			
Spontaneous, 2260	1648(72.9%)	612(27.1%)	
Instrumental, 288	191(66.3%)	97(33.7%)	0.021
Intact cord during stimulation			
No, 1892	1303(68.9%)	589(31.1%)	
Yes, 671	544(81.1%)	127(18.9%)	<0.0001
Drying after birth			
No, 159	100(62.9%)	59(37.2%)	
Yes, 2404	1747(72.7%)	657(27.3%)	0.01
Gestational age in weeks (SD)	38.53±2.0	38.2±2.7	
Preterm birth (<37 weeks)			
No, 2272	1657 (72.9%)	615 (27.1%)	
Yes, 291	190 (65.3%)	101 (34.7%)	0.008
Birth weights in gram (SD)	2836.9±523.9	2707.6±614.0	
Low Birth Weight (<2500 gram)			
No, 2083	1543 (74.1%)	540 (25.9%)	
Yes, 463	298 (64.3%)	165 (35.7%)	<0.0001

In bivariate analysis, there were 1.94 higher odds of breathing if the cord was intact during stimulation compared with neonates with cord clamped (crude odds ratio (cOR), 1.94; 95% CI: 1.56 to 2.40). If the neonate’s mother had complications during admission for delivery, the odds of breathing after stimulation were 0.50 lower than neonate’s whose mother had no complication during admission (cOR, 0.50; 95% CI: 0.38 to 0.64). Neonates who were dried immediately after birth had 1.57 higher odds of breathing after stimulation than those who were not dried after birth (cOR, 1.57; 95% CI: 1.12 to 2.19). In neonates with birth weight <2500 g, the odds of breathing after stimulation was 0.63 lower than neonate’s with low birth weight (cOR, 0.63; 95% CI: 0.51 to 0.78) (table 4).

**Table 4:** Bivariate and multivariate analysis of association between obstetrics and neonatal characteristics with breathing after stimulation

	Bivariate analysis			Multivariate analysis		
	Beta coefficient	P value	cOR (95% CI)	Beta coefficient	P value	aOR (95% CI)
Global intercept				0.225		1.252
intact cord (reference cord clamped)						
Intercept	0.133		1.143			
Intact cord	0.661	<0.0001	1.94 (1.56 to 2.40)	0.613	<0.0001	1.85 (1.48 to 2.30)
Complication during admission						
Intercept	1.034		2.814			
Yes	−0.701	<0.0001	0.50 (0.38 to 0.64)	−0.572	<0.0001	0.57 (0.43 to 0.75)
Mode of delivery (reference spontaneous birth)						
Intercept	1.304	0	3.683			
Instrumental delivery	−0.313	0.019	0.73 (0.56 to 0.95)	−0.324	0.018	0.72 (0.55 to 0.95)
Immediate drying after birth (reference no drying)						
Intercept	0.528	0.001	1.695			
Immediate drying after birth	0.45	0.008	1.57 (1.12 to 2.19)	0.385	0.028	1.47 (1.04 to 2.07)
Low birth weight (reference 2500 g or more)						
Intercept	1.05	0	2.857			
Low birth weight	−0.459	<0.0001	0.63 (0.51 to 0.78)	−0.383	0.003	0.68 (0.53 to 0.88)
Preterm (reference 37 weeks or more)						
Constant	0.991	0	2.694			
Preterm	−0.359	0.006	0.70 (0.54 to 0.90)	0.041	0.80	1.04 (0.76 to 1.43)

aOR, adjusted OR; cOR, crude OR.

In multivariate analysis, to assess the association of characteristics with breathing after stimulation, there was 1.84 higher odds of breathing if the cord was intact during stimulation when compared with neonates with cord clamped (adjusted OR (aOR), 1.85; 95% CI: 1.48 to 2.30) after adjusting for obstetrical complications during admission, mode of delivery, immediate drying after birth, low birth weight and preterm birth. Neonates who were dried immediately after birth had 1.49 higher odds of breathing after stimulation than those who were not dried after birth (aOR, 1.47; 95% CI: 1.04 to 2.07) (table 4).

## Discussion

In this observational study, one-fourth of the non-crying neonates had stimulation performed with an intact cord. There were higher odds of spontaneous breathing if the cord was kept intact during stimulation than neonates who had their cord clamped during stimulation. The higher obstetrical complications during admission and Apgar Score less than 4 at 1 min among early cord clamped non-crying neonates indicates that these are more depressed neonates than neonates with cord intact. In line with this, a higher proportion of infants with early cord clamping received bag-and-mask ventilation. Even though there was no significant difference in mortality between the two groups, the mortality trended lower among the neonates who were stimulated with the cord intact.

An experimental animal study by Bhatt *et al* demonstrated a more stable cardiovascular adaptation if cord clamping was performed after initiation of ventilation.^7^ The results from this and other studies suggested that resuscitation of newborns should be performed with an uncut cord, facilitating the postnatal transition.^17–19^ However, in a single-centre observation study in Tanzania, there was no significant association between time to cord clamping and onset of breathing or initiation of positive-pressure ventilation following stimulation/suction.^20^

Similar to our results, a single-centre randomised controlled trial (RCT) in California, USA, to evaluate the effect of delayed cord clamping in premature infants (<32 weeks) who required ventilation demonstrated that provision of gentle tactile stimulation with the cord intact may hasten the establishment of spontaneous respirations and provide a similar placental transfusion compared with positive-pressure ventilation with delayed cord clamping.^21^ Another multicentric trial in the UK among premature infants (≤32 weeks) suggests that cord clamping after at least 2 min and providing neonatal care with the cord intact may improve outcome at discharge as compared with early clamping.^22^

A single-centre RCT in Nepal evaluated the effect on delayed versus early cord clamping in babies requiring resuscitation.^23^ In the trial, randomisation was performed while the baby was still in utero and resuscitation measures (stimulation and positive-pressure ventilation) performed according to the HBB algorithm with an unclamped cord in the mother’s bed were associated with early spontaneous breathing.

Our multicentric study conducted between 2017 and 2018 showed that 9.8% of neonates do not cry at birth.^12^ The study found that non-crying but breathing babies might not have the full respiratory capacity and require resuscitation.^24^ In this study, we hypothesise that the non-crying neonates who breathe spontaneously after stimulation might have been in primary apnea. There is increased oxygenation with intact cord due to several interacting mechanisms: persisting oxygenation by the placenta, avoidance of bradycardia often associated with clamping the cord before the onset of respirations and earlier initiation of breathing resulting in increased pulmonary blood flow.

A study in Tanzania showed that although the healthcare provider commonly practiced clamping the umbilical cord immediately after delivery, they were aware that delayed cord clamping has a potential benefit of oxygenation to the newborn in the event of the need for resuscitation.^25^ Our study also shows that despite the education of healthcare providers on keeping the cord intact during resuscitation, two-thirds of the non-crying babies received resuscitation after cord clamping. There is a need to implement a delay in clamping as a quality improvement intervention to improve initial steps of resuscitation—especially stimulation and if necessary bag-and-mask ventilation. Three recent systematic reviews and meta-analysis showed that there is a lack of evidence to recommend cord management among term and preterm infants who receive positive-pressure ventilation at birth.^26 27^

### Methodological consideration

This study has several strengths including that observations were done by trained researchers in multiple hospitals in Nepal in a consistent way and with large sample size. Using observational methods limited recall bias. However, there are several limitations. First, this observation was only done for vaginal births and not for caesarean births. Second, cord management was at the discretion of the health workers, so there may have been a bias toward the previous practice of immediate cord clamping. The appearance of the baby at birth (degree of cyanosis, hypotonia) may have influenced decision-making on when to cut the cord.

In conclusion, our study found that non-crying neonates stimulated with the cord intact were more likely to breathe than those who were stimulated with the cord clamped. Stimulation with cord intact may help establish spontaneous breathing in apnoeic neonates, but residual confounding variables are likely contributing to the findings. Our study provides additional evidence consistent with the ILCOR recommendation on keeping the cord intact during the initial steps of resuscitation. This study highlights the importance of further controlled research to evaluate the effect of initial steps of resuscitation with cord intact.

## Supplementary Material

Author's manuscript

## Data Availability

Data sharing not applicable as no datasets were generated and/or analysed for this study. Data are available upon reasonable request. The dataset generated and analysed is not publicly available as it is part of larger quality improvement projects but can be made available on reasonable request with a data-sharing agreement.

## References

[1] Lee ACC , Cousens S , Wall SN , et al . Neonatal resuscitation and immediate newborn assessment and stimulation for the prevention of neonatal deaths: a systematic review, meta-analysis and Delphi estimation of mortality effect. BMC Public Health 2011;11 Suppl 3:S12. 10.1186/1471-2458-11-S3-S12 PMC3231885 21501429

[2] Hooper SB , Polglase GR , te Pas AB . A physiological approach to the timing of umbilical cord clamping at birth. Arch Dis Child Fetal Neonatal Ed 2015;100:F355–60. 10.1136/archdischild-2013-305703 25540147

[3] Lee ACC , Kozuki N , Blencowe H , et al . Intrapartum-related neonatal encephalopathy incidence and impairment at regional and global levels for 2010 with trends from 1990. Pediatr Res 2013;74 Suppl 1:50–72. 10.1038/pr.2013.206 24366463 PMC3873711

[4] Liu L , Oza S , Hogan D , et al . Global, regional, and national causes of under-5 mortality in 2000-15: an updated systematic analysis with implications for the sustainable development goals. Lancet 2016;388:3027–35. 10.1016/S0140-6736(16)31593-8 27839855 PMC5161777

[5] Gao Y , Raj JU . Regulation of the pulmonary circulation in the fetus and newborn. Physiol Rev 2010;90:1291–335. 10.1152/physrev.00032.2009 20959617

[6] Crossley KJ , Allison BJ , Polglase GR , et al . Dynamic changes in the direction of blood flow through the ductus arteriosus at birth. J Physiol 2009;587:4695–704. 10.1113/jphysiol.2009.174870 19675069 PMC2768022

[7] Bhatt S , Alison BJ , Wallace EM , et al . Delaying cord clamping until ventilation onset improves cardiovascular function at birth in preterm lambs. J Physiol 2013;591:2113–26. 10.1113/jphysiol.2012.250084 23401615 PMC3634523

[8] Polglase GR , Dawson JA , Kluckow M , et al . Ventilation onset prior to umbilical cord clamping (physiological-based cord clamping) improves systemic and cerebral oxygenation in preterm lambs. PLoS One 2015;10:e0117504. 10.1371/journal.pone.0117504 25689406 PMC4331493

[9] Katheria AC , Brown MK , Rich W , et al . Providing a placental transfusion in newborns who need resuscitation. Front Pediatr 2017;5:1. 10.3389/fped.2017.00001 28180126 PMC5263890

[10] Aziz K , Lee CHC , Escobedo MB , et al . Part 5: neonatal resuscitation 2020 American heart association guidelines for cardiopulmonary resuscitation and emergency cardiovascular care. Pediatrics 2021;147. 10.1542/peds.2020-038505E. [Epub ahead of print: 21 10 2020]. 33087555

[11] World Health Organization . Guidelines on basic newborn resuscitation. Geneva: WHO, 2012. 23700652

[12] Kc A , Lawn JE , Zhou H , et al . Not crying after birth as a predictor of not breathing. Pediatrics 2020;145. 10.1542/peds.2019-2719. [Epub ahead of print: 12 05 2020]. 32398327

[13] Kc A , Ewald U , Basnet O , et al . Effect of a scaled-up neonatal resuscitation quality improvement package on intrapartum-related mortality in Nepal: a stepped-wedge cluster randomized controlled trial. PLoS Med 2019;16:e1002900. 10.1371/journal.pmed.1002900 31498784 PMC6733443

[14] Kamath-Rayne BD , Thukral A , Visick MK , et al . Helping babies breathe, second edition: a model for strengthening educational programs to increase global newborn survival. Glob Health Sci Pract 2018;6:538–51. 10.9745/GHSP-D-18-00147 30287531 PMC6172134

[15] Gurung R , Jha AK , Pyakurel S , et al . Scaling up safer birth bundle through quality improvement in Nepal (SUSTAIN)-a stepped wedge cluster randomized controlled trial in public hospitals. Implement Sci 2019;14:65. 10.1186/s13012-019-0917-z 31217028 PMC6582583

[16] Gurung R , Gurung A , Basnet O , et al . Refine (rapid feedback for quality improvement in neonatal rEsuscitation): an observational study of neonatal resuscitation training and practice in a tertiary hospital in Nepal. BMC Pregnancy Childbirth 2020;20:756. 10.1186/s12884-020-03456-z 33272242 PMC7712979

[17] Niermeyer S , Velaphi S . Promoting physiologic transition at birth: re-examining resuscitation and the timing of cord clamping. Semin Fetal Neonatal Med 2013;18:385–92. 10.1016/j.siny.2013.08.008 24055300

[18] Mercer JS , Erickson-Owens DA . Is it time to rethink cord management when resuscitation is needed? J Midwifery Womens Health 2014;59:635–44. 10.1111/jmwh.12206 25297530 PMC4690467

[19] Bjorland PA , Ersdal HL , Eilevstjønn J , et al . Changes in heart rate from 5 S to 5 min after birth in vaginally delivered term newborns with delayed cord clamping. Arch Dis Child Fetal Neonatal Ed 2021;106:311-315. 10.1136/archdischild-2020-320179 33172876 PMC8070647

[20] Ersdal HL , Linde J , Auestad B , et al . Timing of cord clamping in relation to start of breathing or ventilation among depressed neonates-an observational study. BJOG 2016;123:1370–7. 10.1111/1471-0528.13778 26701211

[21] Katheria A , Poeltler D , Durham J , et al . Neonatal resuscitation with an intact cord: a randomized clinical trial. J Pediatr 2016;178:75–80. 10.1016/j.jpeds.2016.07.053 27574999 PMC5527831

[22] Duley L , Dorling J , Pushpa-Rajah A , et al . Randomised trial of cord clamping and initial stabilisation at very preterm birth. Arch Dis Child Fetal Neonatal Ed 2018;103:F6–14. 10.1136/archdischild-2016-312567 28923985 PMC5750367

[23] Andersson O , Rana N , Ewald U , et al . Intact cord resuscitation versus early cord clamping in the treatment of depressed newborn infants during the first 10 minutes of birth (Nepcord III) - a randomized clinical trial. Matern Health Neonatol Perinatol 2019;5:15. 10.1186/s40748-019-0110-z 31485335 PMC6714434

[24] Tingay DG , Farrell O , Thomson J , et al . Imaging the respiratory transition at birth: unraveling the complexities of the first breaths of life. Am J Respir Crit Care Med 2021;204:82–91. 10.1164/rccm.202007-2997OC 33545023

[25] Mwakawanga DL , Mselle LT . Early or delayed umbilical cord clamping? experiences and perceptions of nurse-midwives and obstetricians at a regional referral hospital in Tanzania. PLoS One 2020;15:e0234854. 10.1371/journal.pone.0234854 32569338 PMC7307749

[26] Jeevan A , Ananthan A , Bhuwan M , et al . Umbilical cord milking versus delayed cord clamping in term and late-preterm infants: a systematic review and meta-analysis. J Matern Fetal Neonatal Med 2021:1–11 (published Online First: 2021/02/12). 10.1080/14767058.2021.1884676 33567910

[27] Seidler AL , Gyte GML , Rabe H , et al . Umbilical cord management for newborns <34 weeks' gestation: a meta-analysis. Pediatrics 2021;147. 10.1542/peds.2020-0576 PMC7924139 33632931

