# Supplementary material for: Impact of stimulation among non-crying neonates with intact cord versus clamped cord on birth outcomes: observation study
Source: BMJ Paediatr Open. 2021 Jan 15;5(1):e001207. doi: 10.1136/bmjpo-2021-001207 (PMC8488701; doi:10.1136/bmjpo-2021-001207)
Supplement: Supplementary data [file bmjpo-2021-001207supp001.pdf]

ID NUMBER: ______________________

Form 1

SUSTAIN Registry Data

**Data Collector details**	Name	
	Code	☐☐☐☐☐☐☐☐

Data ID	Information		Write or circle where applicable	Remarks
**PART A: BACKGROUND INFORMATION**				
101.	Mother's first name			
102.	Mothers' last name			
103.	Mother's inpatient number		☐☐☐☐☐☐☐☐	
104.	Age of mother (completed years)		☐☐	
105.	Ethnicity code		Dalit……………………………………………….1<br>Janajati…………………………………………… 2<br>Madhesi……………………………………………..3<br>Muslim………………………………………………4<br>Brahmin/Chhetri…………………………………….5<br>Others………………………………………………..6	
106.	Address	Province		
		District		
		Municipality		
		Ward		
107.	Mobile		☐☐☐☐☐☐☐☐☐☐	
108a	Place of referall for Delivery		Primary health centre…………………..1<br>District Hospital………………………… 2<br>Health post……………………………… 3<br>Other ……………………………………. 4<br>NR………………………………………..99<br>Self……………………………………….5	
108.	Parity		Primipara (0 previous births)………....……………1	
			Multipara (2-5 births)..………………......................2	
			Grand multipara (>5 births)……………......................3	

	Date (AD) dd/mm/yyyy	Signature
Form completed:		
Data entered into database:		

PART B: PRE-DELIVERY DETAILS					
**Part B1: Complications during pregnancy (based on ANC records)**					
109.	**Complications recorded**	***Yes***	***No***	***NR***	
109a.	*Vaginal bleeding/APH*	1	0	99	
109b.	*Decreased foetal movements*	1	0	99	
109c.	*Eclampsia (including convulsions, coma, stroke or unconsciousness)*	1	0	99	
109d.	*Pre-eclampsia (BP >140/9mmHg and protein urine)*	1	0	99	
109e.	*Diabeties*	1	0	99	
109f.	*TORCHES*	1	0	99	
109g	*TORCHES (specify)……………………………*				
109h.	*Others (specify) ……………………………*				
110.	Place making referral				
111.	Anaemia	Yes………………..…………...1<br>No……………………………..…. 2<br>Not recorded……………..……..99			**If No or NR, go to 114**
112.	If Yes, type of anaemia	Mild (<11 gm/dL)………………. 1<br>Moderate (7-10.9 gm/dL)…...2<br>Severe (<7 gm/dL)……………. 3			
113.	Cause of anaemia (multiple choice)	Iron deficiency…………………1<br>Folic acid deficiency…………..2<br>Vitamin B12 deficiency……..3<br>Sickle cell………………………...4<br>Thalassemia…………………...5<br><br>Others (specify)……………..….			
Height	Height of the mother in cm	……………………. CM			
Weight	Weight of the mother from 1st ANC checkup	……………………….KG's			
**Part B2: Condition at the time of admission**					
114.	**Provisional diagnosis of any complication recorded at the time of admission**	***Yes***	***No***	***NR***	
114a.	*Antepartum haemorrhage (APH)*	1	0	99	
114b.	*Decreased foetal movements*				
114c.	*Prolonged labour (>12 hours active phase)*	1	0	99	
114d.	*Pre-eclampsia (BP >140/9mmHg and protein urine)*	1	0	99	
114e.	*Eclampsia (including convulsions, coma, stroke or unconsciousness)*	1	0	99	
114f.	*Breech or transverse lie*	1	0	99	
114g.	*Prolapsed cord*	1	0	99	
114h.	*Chorioamnionitis*	1	0	99	
114i.	*Premature rupture of membrane (PROM)*	1	0	99	
114j.	*Pre-term labour and/or preterm premature rupture of membranes (PPROM)*	1	0	99	

114k.	*Foetal congenital anomaly*	1	0	99	
114l.	*Others (specify):*				
115.	Gestational age at admission by USG	☐☐ + ☐<br>Not known…………………........ 0<br>Not recorded……………............99			
115A	Gestational age at admission by LMP	☐☐ + ☐☐<br>Not known…………………........ 0<br>Not recorded……………............99			
Q115B	Anemia diagnosed during admission	Yes……………..…………....1<br>No……………………………...... 2<br>Not recorded…………..…..99			
Q115C	If Yes, type of anaemia	Mild (<11 gm/dL)……………. 1<br>Moderate (7-10.9 gm/dL)…..2<br>Severe (<7 gm/dL)…………. 3			
Q115D	Cause of anaemia (multiple choice)	Iron deficiency………………...1<br>Folic acid deficiency………..2<br>Vitamin B12 deficiency……..3<br>Sickle cell…………………...4<br>Thalassemia……………….5<br><br>Others (specify)……………. 6<br>recorded ………………….99			
116.	Mother's blood group	RH positive…………………………..1<br>RH negative……………………….2<br>Not recorded……........................99			
117.	ABO blood type	A…………………………………… 1<br>B…………………………………… 2<br>AB………………………………….3<br>O………………………………….4			
118.	FHR at admission	Yes, normal (100-160 bpm)………..1<br>Yes, abnormal (<110 or >160 bpm)..2<br>Absent…...………….…..............3<br>Not recorded……........................99			**If No or NR, go to 120**
119.	If FHR recorded	Date (AD) dd/mm/yyyy	☐☐☐☐☐☐☐☐		
		Time (hh:mm) 24-hr	☐☐☐☐		
119B	Total Number of PV examinations				
120.	Stage of labour	Not in labour………………….….1<br>Latent stage of labour…………........2<br>First stage of active labour…..........3<br>Second stage of labour……...........4<br>Third stage of labour………...........5			
119A	Duration of active stage of labor	………………… hours			

PART C: DELIVERY DETAILS

121.	Place of delivery	Home……………………..….....1<br>On the way ……………………......2<br>Health facility………....……...3	**If Home or On the way, go to 129**
Q121a	If Health facility	Inside delivery room……………..1<br>Outside delivery room………… 2<br>NR………………………………..99	
122.	Partograph use	Yes, completely filled…………….1<br>Yes, partially filled……………..….2<br>Not filled…………………………….0	
123.	FHR monitoring recorded during delivery	Yes, as per protocol..…………...…1<br>Yes, sporadically (> once)…………2<br>Yes, only once………..…………...3<br>Not recorded……………........99	
124.	Abnormal FHR detected during labour	Yes, bradycardia (< 110)…………..1<br>Yes, tachycardia (> 160)………….2<br>Yes, repetitive (during > 50% of contractions) or prolonged (> 3 min) decelerations……………….3<br>No…………………..……………...0<br>Not recorded…………………...99	
125.	Induction of labour	Induction with prostaglandins…....1<br>Induction with amniotomy………2<br>Induction with oxytocin…………..3<br>No…………………..……………...0<br>Not recorded…………………...99	
126.	Augmentation of labour	Augmentation with oxytocin…….1<br>Augmentation with amniotomy....2<br>No…………………..……………...0<br>Not recorded…………………...99	

127.	Received prophylactic antibiotics	Yes…………………..…………...………1<br>No………………………….......................0	
127A	Corticosteroids given to mother<br><br>(Dexamethasone/Betamethasone)	Yes…………………..…………...………1<br>No………………………….......................0	
128.	Mode of delivery	Spontaneous vaginal…………...…………1<br>***Go to 131***<br><br>Instrumental………...………..…............2<br>***Go to 130***<br><br>Manoeuvre delivery…………………………...3<br>***Go to 131***<br><br>Emergency CS…………………………......4<br>Elective CS………..………...............……….5<br>***Go to 129***	

129.	Reason for CS (multiple response)	Foetal distress (FD)………………………..……..1<br>Cephalopelvic disproportion (CPD)………….....2<br>Abnormal lie/malpresentation/ malposition…3<br>APH or intrapartum haemorrhage …………..4<br>PIH (Pre-eclampsia/eclampsia)……………..….5<br>Non-progress of labour (NPOL) or prolonged labour……………………………………..….…...…..6<br>Multiple pregnancy……….......................…......7<br>Cord around the neck…………………………..8<br>Cord prolapse………………………..…………...9<br>Oligohydramnios…………………...…………..10<br>Previous CS…...…………………...........….....11<br>Maternal request…………………………..…......12<br><br>Other (specify)……………………..………..…….	
130.	Reason for instrumental delivery	Prolonged labour………………..…………….…...1<br>Foetal distress……….……………………..……...2<br>Maternal distress……………………..……….….3<br><br>Other (specify)…...…………….....……………..….	
131.	Multiple delivery	Yes……………………..………………..………..1<br>No…………………………………......................0	
If multiple deliveries, use additional form for each baby!			
132.	Delivery conducted by	Nursing staff…………………………………..….…1<br>Doctor……………………………..……………..…2<br>Other health personnel……..…………………….3<br>Not recorded………………………………………..99	
133.	Complication to mother before or at the time of delivery	Yes…………………………………..………..……..1<br>No…………………………………..……..………...0<br>Not recorded…………………………………..…...99	**If No or NR, go to 135**
134.	If Yes (multiple choice)	Manoeuvre delivery………………………..………1<br>Prolonged labour……………………………………2<br>Antepartum haemorrhage…………………………3<br>Postpartum haemorrhage…………………………4<br>Pregnancy induced hypertension.. …………....5<br>Oligohydramnios……………………………………6<br><br>Others (specify)………….… ………….....…….	

135.	Amniotic fluid	Clear………………………………..….….1<br>Thin meconium stained……………....…..2<br>Thick meconium stained……….......…..3<br>Not recorded…………….....………...99	
136.	Complication to baby at the time of delivery	Yes……………………………………..……….1<br>No……………………………..….……………...0<br>Not recorded…………….....………...99	**If No or NR, go to 138**
137.	If Yes (multiple choice)	Birth asphyxia……………………..…………...1<br>Grunting………………...……………..….….2<br>Foetal distress………....…...…...….3<br>Preterm………….....……...………….….4<br>Meconium stained………………..........5<br>Tachypnoea………....…...…………..….6	

		Breech delivery……………..…..…..7<br>Low birth weight…………….…..…8<br>Sepsis………………..……………….9<br>Cyanosis…………..………………10<br>Poor cry………………….……...11<br>Respiratory distress syndrome…...12<br>Big baby…………………………...13<br>Gasping ……………………..…..…...14<br>Others (specify)……………..…….…			
138.	Date of delivery (AD) dd/mm/yyyy	☐☐☐☐☐☐☐☐			
139.	Time of delivery (hh:mm) 24-hr	☐☐☐☐			
140.	Sex of baby	Girl………………………........…....1<br>Boy……………….........................0			
141.	Birth weight (grams)	☐☐☐☐			
142.	Gestational age by LMP (weeks)	☐☐ + ☐<br>Not known…………………........ 0<br>Not recorded……………............99			
143.	Delivery outcome	Livebirth……………..……….......1<br>Stillbirth……....…………………...2			**If Livebirth, go to 145**
144.	If stillbirth, type of stillbirth	Fresh………………………….…..…1<br>Macerated………………………......... 2			**End extraction!!!**
145.	APGAR at 1 minute	☐☐			
146.	APGAR at 5 minutes	☐☐			
147.	**Method of Resuscitation**	***Yes***	***No***	***NR***	
147a.	Clearing of airway (suctioning)	1	0	99	
147b.	Stimulation	1	0	99	
147c.	Bag-mask ventilation	1	0	99	
	Other interventions				
147d.	Oxygen	1	0	99	
147e.	Medication	1	0	99	
147f.	Chest compression	1	0	99	
147g.	Intubation	1	0	99	
147h.	Others (specify)…………………………	1	0	99	
148.	Malformation	1	0	99	**If No or NR, go to 150**
149.	If Yes (multiple response)	Neural tube defects……………….1<br>Cleft lip……….……………………2<br>Cleft palate……………………….3<br>Club foot………………………….4<br>Hypospadias………………………5<br>Omphalocele……………....……6<br>Gastroschisis……………………7<br>Imperforate anus…………………8<br>Other limb defects………………..9<br><br>Others (specify)……………………			
	Routine care of newborn	***Yes***	***No***	***NR***	
150.	Vitamin K	1	0	99	

151.	Body temperature	1	0	99	
152.	Respiratory rate	1	0	99	
153.	Medical examination of baby	1	0	99	
154.	Newborn transferred from labour room	Yes, SNCU/NICU………………......1<br>Yes, PNC……………………………..2<br>No………..…………………..….0<br>Not recorded……..…………........99			**If SNCU/NICU, go to Part D**
155.	Outcome of the baby	Healthy…………………………….1<br>Improved…………………………....2<br>Referred to other facility……..….…3<br>**Died……..…………………………4**<br>Absconded………………....……..5<br>Stillbirth……………………………..7<br>DOPR………………………………..8			**If other than died goto q158**
156.	Date of death	☐☐☐☐☐☐☐☐			
157.	Cause of death (multiple response)	Neonatal sepsis/infection…………1<br>Meconium aspiration syndrome….2<br>Birth asphyxia………………………..3<br>Respiratory distress syndrome…..4<br>Hypoglycaemia……………………5<br>Low birth weight (LBW)………….6<br>Preterm……………………………7<br>Congenital malformation………..8<br><br>Others (specify)………………………..			
158.	Date of discharge (AD) dd/mm/yyyy	☐☐☐☐☐☐☐☐			
