# Supplementary material for: Impact of stimulation among non-crying neonates with intact cord versus clamped cord on birth outcomes: observation study
Source: BMJ Paediatr Open. 2021 Jan 15;5(1):e001207. doi: 10.1136/bmjpo-2021-001207 (PMC8488701; doi:10.1136/bmjpo-2021-001207)
Supplement: Author's manuscript [file bmjpo-2021-001207.draft_revisions.pdf]

BMJ Paediatrics Open

BMJ Paediatrics Open is committed to open peer review. As part of this commitment we make the peer review history of every article we publish publicly available.

When an article is published we post the peer reviewers' comments and the authors' responses online. We also post the versions of the paper that were used during peer review. These are the versions that the peer review comments apply to.

The versions of the paper that follow are the versions that were submitted during the peer review process. They are not the versions of record or the final published versions. They should not be cited or distributed as the published version of this manuscript.

BMJ Paediatrics Open is an open access journal and the full, final, typeset and author-corrected version of record of the manuscript is available on our site with no access controls, subscription charges or pay-per-view fees (http://bmjpaedsopen.bmj.com).

If you have any questions on BMJ Paediatrics Open's open peer review process please email info.bmjpo@bmj.com

BMJ Paediatrics Open

Impact of stimulation among non-crying neonates with intact cord versus clamped cord on birth outcomes-observation study

Journal:	*BMJ Paediatrics Open*
Manuscript ID	bmjpo-2021-001207
Article Type:	Original research
Date Submitted by the Author:	28-Jun-2021
Complete List of Authors:	KC, Ashish; Uppsala University, Department of Women's and Children's Health<br>Budhathoki, Shyam; Imperial College London, Department of Primary Care and Public Health; Golden Community<br>Thapa, Jeevan; Patan Academy of Health Sciences, Department of Community Health Sciences<br>Niermeyer, Susan; University of Colorado Denver School of Medicine, Pediatrics<br>Gurung, Rejina; Uppsala University, Department of Women's and Children's Health; Golden Community<br>Singhal, Nalini; University of Calgary, Pedaitrics
Keywords:	Resuscitation, Epidemiology, Neonatology

SCHOLARONE™
Manuscripts

I, the Submitting Author has the right to grant and does grant on behalf of all authors of the Work (as defined in the below author licence), an exclusive licence and/or a non-exclusive licence for contributions from authors who are: i) UK Crown employees; ii) where BMJ has agreed a CC-BY licence shall apply, and/or iii) in accordance with the terms applicable for US Federal Government officers or employees acting as part of their official duties; on a worldwide, perpetual, irrevocable, royalty-free basis to BMJ Publishing Group Ltd ("BMJ") its licensees and where the relevant Journal is co-owned by BMJ to the co-owners of the Journal, to publish the Work in this journal and any other BMJ products and to exploit all rights, as set out in our licence.

The Submitting Author accepts and understands that any supply made under these terms is made by BMJ to the Submitting Author unless you are acting as an employee on behalf of your employer or a postgraduate student of an affiliated institution which is paying any applicable article publishing charge ("APC") for Open Access articles. Where the Submitting Author wishes to make the Work available on an Open Access basis (and intends to pay the relevant APC), the terms of reuse of such Open Access shall be governed by a Creative Commons licence – details of these licences and which Creative Commons licence will apply to this Work are set out in our licence referred to above.

Other than as permitted in any relevant BMJ Author's Self Archiving Policies, I confirm this Work has not been accepted for publication elsewhere, is not being considered for publication elsewhere and does not duplicate material already published. I confirm all authors consent to publication of this Work and authorise the granting of this licence.

Confidential: For Review Only

Impact of stimulation among non-crying neonates with intact cord versus clamped cord on birth outcomes- observation study

Ashish KC[1], Shyam Sundar Budhathoki[2,3], Jeevan Thapa[4], Susan Niermeyer[5], Rejina Gurung[1,3], Nalini Singhal[6] on behalf of Nepal Neonatal Network[ø]

[1]Department of Women's and Children's Health, Uppsala University, Uppsala, Sweden;

[2]Department of Primary Care and Public Health, School of Public Health, Imperial College London, London, United Kingdom;

[3]Golden Community, Lalitpur, Nepal;

[4] Department of Community Health Sciences, Patan Academy of Health Sciences, Lalitpur, Nepal;

[5]University of Colorado School of Medicine, Colorado School of Public Health, Aurora, CO, USA;

[6]Department of Pediatrics, University of Calgary, Calgary, Canada.

Correspondence- Ashish KC, Department of Women's and Children's Health, Uppsala University, SE-751 85, Uppsala, Sweden, www.kbh.uu.se/imch. Email address: ashish.k.c@kbh.uu.se; Phone number: +46 0729001074

Confidential: For Review Only

ABSTRACT

Background

Stimulation of non-crying neonates after birth can help transition to spontaneous breathing. In this study, we aim to assess the impact of intact versus clamped umbilical cord on spontaneous breathing after stimulation of non-crying neonates.

Methods

This is an observational study among non-crying neonates (n=3,073) born in hospitals of Nepal. Non-crying neonates born vaginally at gestational age ≥34-weeks were observed for their response to stimulation with the cord intact or clamped. Obstetric characteristics of the neonates were analysed. Association of spontaneous breathing with cord management was assessed using logistic regression.

Results

Among non-crying neonates, 2,563 of them received stimulation. Of these, a higher proportion of the neonates were breathing in the cord intact group compared to those cord clamped group (81.1% vs 68.9%, p<0.0001). The use of bag-and-mask ventilation was lower among those who were stimulated with the cord intact than those who were stimulated with cord clamped (18.0% vs 32.4%, p<0.0001). The proportion of neonates with Apgar-score ≤3 at 1 minute was lower with the cord intact than with cord clamped (7.6% vs 11.5%, p-0.001). In multi-variate analysis, neonates with intact cord had 84%

Confidential: For Review Only

increased odds of spontaneous breathing (aOR, 1.84; 1.48, 2.29) compared to those with cord clamped.

Conclusions

Stimulation of non-crying neonates with intact cord was associated with more spontaneous breathing than who were stimulated with cord clamped. This indicates that primary apnoeic neonate transition better when stimulated with cord intact. This study provides evidence potential benefits to performing the initial steps of resuscitation with the cord intact.

Key words: non-crying neonates, stimulation with cord intact, spontaneous breathing, resuscitation.

Confidential: For Review Only

What is already known on this topic?

- Stimulation is one of the key steps to neonatal resuscitation
- Breathing prior to umbilical cord clamping has been shown to result in smoother cardiovascular transition at birth in experimental studies

What this study adds?

- Stimulation with the cord intact may be more effective in triggering spontaneous breathing in primary apnoeic neonates than stimulation provided after cord clamping.

Confidential: For Review Only

INTRODUCTION

Globally, every year among the 140 million neonates who are born, 10-15 million do not cry or breathe at birth[1]. These babies require resuscitation to transition from the intrauterine to the extra-uterine environment[2]. Neonates who do not receive timely and adequate resuscitation may die or suffer brain injuries and long-term disability[3]. Every year, an estimated 1 million neonates die due to intrapartum related complications or "birth asphyxia"[4], 2 million experience hypoxic-ischemic encephalopathy and 1.2 million go on to show developmental delay[3]. Most of this mortality and morbidity occur in low- and middle-income countries.

Transitioning to an extra-uterine environment depends on two major physiological events, the commencement of breathing and the transition of blood flow from the umbilical to the pulmonary circulation[2 5]. The trigger of breathing at birth results in absorption of the liquid in the trachea and airways, aeration of the lungs, reduction in pulmonary vascular resistance, and increase in pulmonary blood flow[6 7]. This increase in pulmonary blood flow supplies left ventricular preload previously dependent on flow across the patent foramen ovale from the right atrium[2]. Any intrapartum insult due to placental insufficiency (hypertension, infection or haemorrhage) can delay the spontaneous cardiopulmonary transition and may be compounded by umbilical cord clamping [8 9]. As a result, blood flows continuously through the ductus arteriosus, by-passing the pulmonary circulation into the systemic circulation as right to left shunting[6]. Thus, resuscitation with the cord intact has been recommended.

Confidential: For Review Only

The International Liaison Committee on Resuscitation provides guidance on neonatal resuscitation[10]. The ILCOR 2020 recommends that a neonate who is not crying or breathing with poor tone and heart rate less than 100 bpm should receive stimulation and clearing of airways (as needed) with intact cord[10]. There is very little robust evidence on the impact of stimulation in neonates receiving resuscitation with the cord intact. The World Health Organization (WHO) recommends if there is an experience in providing ventilation without cutting the umbilical cord, ventilation can be initiated before cord-cutting.[11] WHO identifies resuscitation with the cord intact as a key research area to generate a stronger evidence base for care.[11].

Studies from low and middle-income countries have reported that neonates who are not crying or breathing at birth are immediately taken to a neonatal resuscitation area for stimulation, clearing of airways and positive-pressure ventilation[12 13]. Despite educational programs such as Helping Babies Breathe (HBB) from the American Academy of Pediatrics, which advocates stimulation to breathe before clamping the cord, health workers often clamp the cord before initiating resuscitation [14].

This study aims to evaluate the impact of stimulation among neonates with the cord intact versus those with cord clamped on breathing and birth outcome.

METHODS:

Study Design: This was a nested observational study within two quality improvement studies SUSTAIN[15] and REFINE[16] done in nine hospitals of Nepal.

Confidential: For Review Only

Study Sites: This study was conducted among nine hospitals in Nepal. The hospitals are tertiary care hospitals providing referral obstetric services through Comprehensive and Emergency Obstetric and Neonatal Care Services. These hospitals were chosen for those quality improvement studies to implement a safer birth bundle package. Health workers were trained on HBB 2.0 to resuscitate neonates with intact cord where possible as part of the safer birth bundle. The cord management was at the discretion of the individual health worker.

Study Dates: The study was conducted between 1 June 2019 to 2 May 2020.

Participants: Women in labour with gestational age ≥34 weeks and confirmed fetal heart rate during admission were considered eligible for the study and approached for consent. Those women who underwent caesarean section for delivery, or whose birth were not observed were excluded from the study. Non-crying newborns who received additional tactile stimulation were included in the study. Furthermore, the babies who cried immediately after birth and those with birth defects were excluded from the study. Neonates who did not cry were included in the analysis.

Sample size: All non-crying neonates who received tactile stimulation were enrolled were considered for analysis.

Variables: The variables considered were defined as cord status: intact and clamped; Parity: no previous birth, 1 previous birth and 2 or more previous births; maternal complication during admission: any medical/obstetric complications during admission of the women for labour, induction of labour, augmentation of labour; mode of

https://mc.manuscriptcentral.com/bmjpo

Confidential: For Review Only

delivery: instrumental birth (for forceps, vacuum application) or spontaneous; preterm birth (age of gestation less than 37 weeks); low birth weight (weight of baby less than 2500 gm); immediate thorough drying after birth and breathing after stimulation (spontaneous breathing requiring no positive pressure ventilation). Stillbirth defined as no heart rate or breathing after resuscitation action.

Data collection and Management: The data collection was done through the existing data collection systems of the two studies REFINE and SUSTAIN. Each of the participating hospitals had systems for individual-level data collection. Observational data was collected by independent clinical researchers using a tablet-based application. They also collected obstetric and neonatal information for the patient case notes. They recorded information on induction, augmentation of labour, complications during admission (antepartum haemorrhage, hypertensive disorder, premature rupture of member, abnormal presentation, infection), mode of delivery (spontaneous and instrumental vaginal), sex and weight of baby, crying status of baby, and whether drying and clearing of the airway were performed. An independent database manager reviewed the data entered in the tablet-based application weekly. The data was then extracted into SPSS software for cleaning, coding and analysis.

Data Analysis: The non-crying babies included in the study were categorised as those who were stimulated with intact cord and those who were stimulated after cord clamping. The background characteristics among these two groups were compared. A comparison was done for obstetric and neonatal characteristics.

Confidential: For Review Only

Spontaneous breathing after stimulation was observed among these two groups to see the impact of intact versus clamped cord at the time of stimulation. The multivariable analysis using logistic regression was done to assess the association of cord status during stimulation on breathing by adjusting for the variables which had a significant level difference of p<=0.01. The variables which were significantly different in cord status during stimulation were complications during admission, mode of delivery and immediate drying.

Patients and public involvement- The main study aimed to improve the provision and experience of care of patients (mother and newborn) during childbirth. This study was based on the patient's experience to improve care after birth, so that patient's receive better care at birth. Information were provided to patients on their involvement in the main study. Dissemination of the result from this study will be done through local dissemination and usefulness of stimulation of non-crying neonates with cord intact.

Ethical Approval: Ethical approval was granted by the Ethical Review Board of the Nepal Health Research Council for SUSTAIN (ref. 110/2019) and REFINE studies (ref. 87/2018). Written informed consent was obtained from the participants at the time of admission to the hospital before enrolling in the study.

RESULTS:

During the study period, 41,621 women delivered in the hospitals and 34,652 women consented to the study. Among the consented women 27,652 births were observed in

Confidential: For Review Only

the study. Among the total births observed 24,071 babies were excluded as they cried immediately after birth, and 508 births were further excluded due to birth defects; 3073 non-crying neonates were included in the analysis. Among the non-crying neonates, 2563 were stimulated. Of the neonates stimulated, 671 (26.2%) of them had their cord intact while 1892 (73.8%) had their cord clamped (figure 1). Of the neonates who had the cord intact during stimulation, 81.1% of them had smooth and spontaneous breathing, while 68.9% of the neonates with cord clamped were breathing after stimulation (p-value <0.0001) (figure 2).

We compared the obstetric and neonatal characteristics of neonates who had cord intact versus cord clamped before stimulation. Maternal complication during admission was lower among neonates who had cord intact than those who had the cord clamped (8.0% vs 11.2%, p-value=0.022). The induction of labour was lower among neonates who had their cord intact than those who had the cord clamped (18.5% vs 25.5%, p-value <0.0001). Among neonates who were born through instrumental delivery, the proportion with cord intact was lower than those who had the cord clamped (8.4% vs 12.3%, p-value=0.005) (Table 1).

The clinical outcome differed among neonates stimulated with the cord intact vs with those cord clamped. Among neonates stimulated with the cord intact, 81.1% breathed, while 68.9% breathed after stimulation with cord clamped (p-value <0.0001). The use of bag-and-mask ventilation was lower among those who were stimulated with the cord intact than those who were stimulated with cord clamped (18.0% vs 32.4%, p-

Confidential: For Review Only

value <0.0001). The proportion of neonates with Apgar score 3 or less at 1 minute was higher with the cord intact than those who were cord clamped (92.1% vs 88.3%, p-value 0.001). The proportion of neonates with Apgar score 6 or less at 5 minutes was higher with the cord intact than those who were cord clamped (92.1% vs 87.3% p-value <0.0001). The proportion of mortality was lower among the neonates who had their cord intact but statistically not significantly different from those with cord clamped (0.7 vs 1.6%, p-value 0.123) (Table 2).

We compared the obstetric and neonatal characteristics of non-crying neonates who breathed after stimulation. 58.3% of neonates with a maternal complication during admission breathed after stimulation, while 73.8% of neonates without maternal complication breathed after stimulation (p-value <0.0001). 66.3% of neonates who were born through instrumental delivery breathed after stimulation, while 72.9% of neonates who were born through spontaneous delivery breathed after stimulation (p-value=0.021). 62.9% of neonates who were not immediately dried after birth breathed after stimulation, while 72.7% of neonates who were immediately dried after birth breathed after additional stimulation (p-value=0.01). (Table 3)

In bivariate analysis, there is 1.94 higher odds of breathing if the cord was intact during stimulation compared to the neonates with cord clamped (cOR, 1.94; 95% CI; 1.56, 2.40). If the neonate's mother had complications during admission for delivery, the odds of breathing after stimulation was 0.50 lower than neonate's whose mother had no complication during admission (cOR, 0.50; 95% CI; 0.38, 0.64). Neonates who were

Confidential: For Review Only

dried immediately after birth had 1.57 higher odds of breathing after stimulation than those who were not dried after birth (cOR, 1.57; 95% CI; 1.12, 2.19). In neonates with birth weight 2500 grams, the odds of breathing after stimulation was 0.63 lower than neonate's with low birth weight (cOR, 0.63; 95% CI; 0.51, 0.78). (Table 4)

In multi-variate analysis, to assess the association of characteristics with breathing after stimulation, there was 1.84 higher odds of breathing if the cord was intact during stimulation when compared to neonates with cord clamped (aOR, 1.84; 95% CI; 1.48, 2.29) after adjusting with a maternal complication during admission, mode of delivery, immediate drying after birth, low birth weight and preterm birth. Neonates who were dried immediately after birth had 1.49 higher odds of breathing after stimulation than those who were not dried after birth (aOR, 1.49; 95% CI; 1.06, 2.10). (Table 4)

DISCUSSION

In this observational study, one-fourth of the non-crying neonates had stimulation performed with an intact cord. There was higher odds of spontaneous breathing if the cord was kept intact during stimulation than neonates who had their cord clamped during stimulation. The higher maternal complication during admission and apgar score less than 4 at 1 minute among early cord clamped non-crying neonates indicates that these are more depressed neonates than neonates with cord intact. This was explained by higher proportion of bag-and-mask ventilation among early cord clamped neonates. Even though there was no significant difference in mortality

Confidential: For Review Only

between the two groups, the mortality trended lower among the neonates who were stimulated with the cord intact.

Similar to our results, a single-center randomized controlled trial (RCT) in California, US to evaluate the effect of delayed cord clamping in premature infants (<32 weeks) who required ventilation demonstrated that provision of gentle tactile stimulation with the cord intact may hasten the establishment of spontaneous respirations and provide a similar placental transfusion compared to positive-pressure ventilation with delayed cord clamping.[17] Another multi-centric trial in the UK among premature infants (≤ 32 weeks) suggests that cord clamping after at least 2 min and providing neonatal care with the cord intact may improve outcome at discharge for the infants compared with early clamping and neonatal care after clamping.[18]

A single-center RCT in Nepal evaluated the effect on delayed versus cord clamping in babies requiring resuscitation[19]. In the trial, randomization was performed while the baby was still in utero, and resuscitation measures (stimulation and positive pressure ventilation) performed according to the HBB algorithm with an unclamped cord in the mother's bed were associated with early spontaneous breathing.

In an experimental animal study by Bhatt et al. demonstrated a more stable cardiovascular adaptation if cord clamping was performed after initiation of ventilation[7]. The results from this and other studies suggested that resuscitation of newborns should be performed with an uncut cord, facilitating the postnatal transition[20-22]. However, in a single-center observation study in Tanzania, there was no

https://mc.manuscriptcentral.com/bmjpo

Confidential: For Review Only

significant association between time to cord clamping and onset of breathing or initiation of positive-pressure ventilation following stimulation/suction[23].

Our multi-centric study conducted between 2017-2018 showed that 9.8% of neonates do not cry at birth[12]. The study found that non-crying but breathing babies might not have the full respiratory capacity and as a result required resuscitation.[24] In this study, we hypothesize that the non-crying neonates who breathe spontaneously after stimulation might have been in primary apnea. There is an increased oxygenation with intact cord due to several interacting mechanisms; persisting oxygenation by the placenta, avoidance of bradycardia often associated with clamping the cord before the onset of respirations, and earlier initiation of breathing resulting in increased pulmonary blood flow.

A study in Tanzania showed that although the health care provider commonly practiced clamping the umbilical cord immediately after delivery, they were aware that delayed cord clamping has a potential benefit of oxygenation to the newborn in the event of the need for resuscitation.[25] Our study also shows that despite the education of health care providers on keeping the cord intact during resuscitation, two-thirds of the non-crying babies received resuscitation after cord clamping. There is a need to implement a delay in clamping as a quality improvement intervention to improve initial steps of resuscitation especially stimulation and if possible bag-and-mask ventilation. Three recent systematic reviews and meta-analysis showed that there is a lack of

Confidential: For Review Only

evidence to recommend cord management among term and preterm infants who receive positive-pressure ventilation at birth.[26 27]

Methodological consideration

This study has several strengths including that observations were done by trained researchers in multiple hospitals in Nepal in a consistent way and with large sample size. Using observational methods limited recall bias. However, there are several limitations. First, this observation was only done for vaginal births and not for caesarean births. Second, the discretion of cord management was decided by health workers, so there is a bias toward the pre-existing cord clamping practice despite being trained on keeping the cord intact during stimulation. Finally, we did not have cord gas values to determine if babies who did not cry had perinatal acidosis or asphyxia.

In conclusion, our study found offer non-crying neonates stimulated with the cord intact more like to breathe than those who are stimulated with cord clamped. This approach will provide the added advantage that stimulation provided by a single provider can induce spontaneous respiration with the cord intact in primary apneic neonates and would not require ventilation and additional providers, especially in low-resource settings. Our study provides additional evidence consistent with the ILCOR recommendation on keeping the cord intact during the initial steps of resuscitation. Despite the training on updated neonatal resuscitation to keep the cord intact, still, three-fourths of the neonates who are not crying have their cord clamped. Further

Confidential: For Review Only

research, education and quality improvement interventions are required to improve cord management among neonates who require help to breathe at birth.

ᴓNepal Neonatal Network-Omkar Basnet, Srijana Sharma, Pratikshya Bhattarai, Ankit Acharya, Prof. Anjani Kumar Jha, Dr. Yasho Vardhan Pradhan, Dr. Sushil Nath Pyakurel, Prof. Kiran Bajracharya, Dr. Sheela Verma, Dr. Amit Bhandari, Dr. Anjana KC Thapa, Dr. Krishna Prasad Bista, Jhalak Sharma Paudel, Sangita Khatri, and Leela Paudel.

Author's contribution-AKC and NS conceptualized the study. AKC, JT and SSB conducted the analysis. NS, RG provided feedback to the analysis. AKC, SSB, RG and JT wrote the first draft. NS and SN made the second and final revision to the draft. All authors reviewed and provided feedback on the revised version and agreed to the final version.

Conflict of Interest-We declare no conflict of interest

Consent for Publication-None

Availability of supporting data

The dataset generated and analysed is not publicly available as it is part of larger quality improvement projects but can be made available on reasonable request with a data-sharing agreement.

Competing interests-The authors declare that they have no competing interests.

Funding-No specific funding was available for this study.

Confidential: For Review Only

Acknowledgement-We would like to thank all the data coordinators and surveillance officers, hospital manager, nursing in-charge, all the nurses and support staff working in the sick newborn care units. We thank all the mothers and caregivers of the sick newborns who consented to the study.

Confidential: For Review Only

References

1. Lee AC, Cousens S, Wall SN, et al. Neonatal resuscitation and immediate newborn assessment and stimulation for the prevention of neonatal deaths: a systematic review, meta-analysis and Delphi estimation of mortality effect. *BMC Public Health* 2011;11 Suppl 3:S12. doi: 10.1186/1471-2458-11-S3-S12 [published Online First: 2011/04/29]
2. Hooper SB, Polglase GR, te Pas AB. A physiological approach to the timing of umbilical cord clamping at birth. *Arch Dis Child Fetal Neonatal Ed* 2015;100(4):F355-60. doi: 10.1136/archdischild-2013-305703 [published Online First: 2014/12/30]
3. Lee AC, Kozuki N, Blencowe H, et al. Intrapartum-related neonatal encephalopathy incidence and impairment at regional and global levels for 2010 with trends from 1990. *Pediatr Res* 2013;74 Suppl 1:50-72. doi: 10.1038/pr.2013.206 [published Online First: 2013/12/25]
4. Liu L, Oza S, Hogan D, et al. Global, regional, and national causes of under-5 mortality in 2000-15: an updated systematic analysis with implications for the Sustainable Development Goals. *Lancet* 2016;388(10063):3027-35. doi: 10.1016/S0140-6736(16)31593-8 [published Online First: 2016/11/15]
5. Gao Y, Raj JU. Regulation of the pulmonary circulation in the fetus and newborn. *Physiol Rev* 2010;90(4):1291-335. doi: 10.1152/physrev.00032.2009 [published Online First: 2010/10/21]
6. Crossley KJ, Allison BJ, Polglase GR, et al. Dynamic changes in the direction of blood flow through the ductus arteriosus at birth. *J Physiol* 2009;587(Pt 19):4695-704. doi: 10.1113/jphysiol.2009.174870 [published Online First: 2009/08/14]
7. Bhatt S, Alison BJ, Wallace EM, et al. Delaying cord clamping until ventilation onset improves cardiovascular function at birth in preterm lambs. *J Physiol* 2013;591(8):2113-26. doi: 10.1113/jphysiol.2012.250084 [published Online First: 2013/02/13]
8. Polglase GR, Dawson JA, Kluckow M, et al. Ventilation onset prior to umbilical cord clamping (physiological-based cord clamping) improves systemic and cerebral oxygenation in preterm lambs. *PLoS One* 2015;10(2):e0117504. doi: 10.1371/journal.pone.0117504 [published Online First: 2015/02/18]
9. Katheria AC, Brown MK, Rich W, et al. Providing a Placental Transfusion in Newborns Who Need Resuscitation. *Front Pediatr* 2017;5:1. doi: 10.3389/fped.2017.00001 [published Online First: 2017/02/10]
10. Aziz K, Lee CHC, Escobedo MB, et al. Part 5: Neonatal Resuscitation 2020 American Heart Association Guidelines for Cardiopulmonary Resuscitation and Emergency Cardiovascular Care. *Pediatrics* 2021;147(Suppl 1) doi: 10.1542/peds.2020-038505E [published Online First: 2020/10/23]
11. Organization WH. Guidelines on basic newborn resuscitation. In: WHO, ed. Geneva: WHO, 2012.
12. Kc A, Lawn JE, Zhou H, et al. Not Crying After Birth as a Predictor of Not Breathing. *Pediatrics* 2020;145(6) doi: 10.1542/peds.2019-2719 [published Online First: 2020/05/14]
13. Kc A, Ewald U, Basnet O, et al. Effect of a scaled-up neonatal resuscitation quality improvement package on intrapartum-related mortality in Nepal: A stepped-wedge cluster randomized controlled trial. *PLoS Med* 2019;16(9):e1002900. doi: 10.1371/journal.pmed.1002900 [published Online First: 2019/09/10]
14. Kamath-Rayne BD, Thukral A, Visick MK, et al. Helping Babies Breathe, Second Edition: A Model for Strengthening Educational Programs to Increase Global Newborn Survival. *Glob Health Sci Pract* 2018;6(3):538-51. doi: 10.9745/GHSP-D-18-00147 [published Online First: 2018/10/06]
15. Gurung R, Jha AK, Pyakurel S, et al. Scaling Up Safer Birth Bundle Through Quality Improvement in Nepal (SUSTAIN)-a stepped wedge cluster randomized controlled trial in public hospitals. *Implement Sci* 2019;14(1):65. doi: 10.1186/s13012-019-0917-z [published Online First: 2019/06/21]

https://mc.manuscriptcentral.com/bmjpo

Confidential: For Review Only

16. Gurung R, Gurung A, Basnet O, et al. REFINE (Rapid Feedback for quality Improvement in Neonatal rEsuscitation): an observational study of neonatal resuscitation training and practice in a tertiary hospital in Nepal. *BMC Pregnancy Childbirth* 2020;20(1):756. doi: 10.1186/s12884-020-03456-z [published Online First: 2020/12/05]
17. Katheria A, Poeltler D, Durham J, et al. Neonatal Resuscitation with an Intact Cord: A Randomized Clinical Trial. *J Pediatr* 2016;178:75-80 e3. doi: 10.1016/j.jpeds.2016.07.053 [published Online First: 2016/10/30]
18. Duley L, Dorling J, Pushpa-Rajah A, et al. Randomised trial of cord clamping and initial stabilisation at very preterm birth. *Arch Dis Child Fetal Neonatal Ed* 2018;103(1):F6-F14. doi: 10.1136/archdischild-2016-312567 [published Online First: 2017/09/20]
19. Andersson O, Rana N, Ewald U, et al. Intact cord resuscitation versus early cord clamping in the treatment of depressed newborn infants during the first 10 minutes of birth (Nepcord III) - a randomized clinical trial. *Matern Health Neonatol Perinatol* 2019;5:15. doi: 10.1186/s40748-019-0110-z [published Online First: 2019/09/06]
20. Niermeyer S, Velaphi S. Promoting physiologic transition at birth: re-examining resuscitation and the timing of cord clamping. *Semin Fetal Neonatal Med* 2013;18(6):385-92. doi: 10.1016/j.siny.2013.08.008 [published Online First: 2013/09/24]
21. Mercer JS, Erickson-Owens DA. Is it time to rethink cord management when resuscitation is needed? *J Midwifery Womens Health* 2014;59(6):635-44. doi: 10.1111/jmwh.12206 [published Online First: 2014/10/10]
22. Bjorland PA, Ersdal HL, Eilevstjonn J, et al. Changes in heart rate from 5 s to 5 min after birth in vaginally delivered term newborns with delayed cord clamping. *Arch Dis Child Fetal Neonatal Ed* 2020 doi: 10.1136/archdischild-2020-320179 [published Online First: 2020/11/12]
23. Ersdal HL, Linde J, Auestad B, et al. Timing of cord clamping in relation to start of breathing or ventilation among depressed neonates-an observational study. *BJOG* 2016;123(8):1370-7. doi: 10.1111/1471-0528.13778 [published Online First: 2015/12/25]
24. David G Tingay, Olivia Farrell, Jessica Thomson, et al. Imaging the Respiratory Transition at Birth: Unravelling the Complexities of the First Breaths of Life. *American Journal of Respiratory and Critical Care Medicine* 2021 February (accepted)
25. Mwakawanga DL, Mselle LT. Early or delayed umbilical cord clamping? Experiences and perceptions of nurse-midwives and obstetricians at a regional referral hospital in Tanzania. *PLoS One* 2020;15(6):e0234854. doi: 10.1371/journal.pone.0234854 [published Online First: 2020/06/23]
26. Jeevan A, Ananthan A, Bhuwan M, et al. Umbilical cord milking versus delayed cord clamping in term and late-preterm infants: a systematic review and meta-analysis. *J Matern Fetal Neonatal Med* 2021:1-11. doi: 10.1080/14767058.2021.1884676 [published Online First: 2021/02/12]
27. Seidler AL, Gyte GML, Rabe H, et al. Umbilical Cord Management for Newborns <34 Weeks' Gestation: A Meta-analysis. *Pediatrics* 2021;147(3) doi: 10.1542/peds.2020-0576 [published Online First: 2021/02/27]

Confidential: For Review Only

Tables and figures

Table 1. Obstetric and neonatal characteristics among non-crying neonates with intact cord versus cord clamped during stimulation

	Intact cord during stimulation n-671	Cord clamped before stimulation n-1892	p-value
Median time to cord clamping (IQR) seconds	58.0 (34, 71)	25 (18, 31)	
Parity			
Nullipara, 1430	376 (56.5%)	1054 (56.0%)	0.892
Primipara, 714	180 (27.0%)	534 (28.4%)	0.515
Multipara, 403	110 (16.5%)	293 (15.6%)	0.579
Complication during admission			
No, 2292	617(92.0%)	1675(88.8%)	
Yes, 266	54 (8.0%)	212 (11.2%)	0.022
Induction of labour			
No, 1957	547 (81.5%)	1410(74.5%)	
Yes, 606	124(18.5%)	482(25.5%)	<0.0001
Augmentation of labour			
No, 1600	445(66.3%)	1155(61.0%)	
Yes, 963	226(33.7%)	737(39.0%)	0.016
Mode of delivery			
Spontaneous, 2260	611(91.6%)	1649(87.7%)	
instrumental, 288	56(8.4%)	232(12.3%)	0.005
Gestational age in weeks (SD)	38.4±2.2	38.5±2.3	
Preterm (<37 weeks)			
No, 2272	594(88.5%)	1678(88.7%)	
Yes, 291	77(11.5%)	214(11.3%)	0.944
Birth weight in weeks (SD)	2784.3±543.7	2807.1±556.6	
Low birth weight (<2500 gram)			
No, 2083	537(82.3%)	1546(82.3%)	
Yes, 463	131(19.6%)	332(17.7%)	0.268
Immediate drying			
No, 159	35(5.2%)	124(6.6%)	
Yes, 2404	636(94.8%)	1892(93.4%)	0.227

Confidential: For Review Only

Table 2. Clinical outcome of non-crying neonates who were stimulated with cord intact vs cord clamped

	Intact cord during stimulation n-671 (26.2%)	Cord clamped before stimulation n-1892 (73.8%)	p-value
Spontaneous breathing			
Yes, 1847	544 (81.1%)	1303 (68.9%)	
No, 716	127 (18.9%)	589 (31.1%)	<0.0001
Bag-and-mask ventilation			
No, 1829	550 (82.0%)	1279 (67.6%)	
Yes, 734	121 (18.0%)	613 (32.4%)	<0.0001
Apgar score at 1 minute			
≤4, 2214	598 (92.4%)	1616 (88.3%)	
≥3, 264	49 (7.6%)	215 (11.5%)	0.001
Apgar score at 5 minute			
≤7, 2196	596 (92.1%)	1597 (87.3%)	
>6, 284	51 (7.9%)	233 (12.7%)	<0.0001
Birth outcome			
Live born, 2513	654 (97.5%)	1859 (98.3%)	
Stillborn, 50	17 (2.5%)	33 (1.7%)	0.198
Mortality			
No, 2528	666 (99.3%)	1862 (98.4%)	
Yes, 35	5 (0.7%)	30 (1.6%)	0.123

Confidential: For Review Only

Table 3. The obstetric and neonatal characteristics of non-crying neonates who responded to stimulation

	Breathing after stimulation (n=1847	Not breathing after stimulation (n=716)	p-value
Complication during admission			
No, 2292	1691(91.6%)	601(84.4%)	
Yes, 266	155(8.4%)	111(15.6%)	<0.0001
Induction of labour			
No, 1957	1406(76.1%)	551(77.0%)	
Yes, 606	441(23.9%)	165(23.0%)	0.679
Augmentation of labour			
No, 1600	1159(62.8%)	441(61.6%)	
Yes, 963	688(37.2%)	275(38.4%)	0.586
Mode of delivery			
Spontaneous, 2260	1648(89.6%)	612(86.3%)	
Instrumental, 288	191(10.4%)	97(13.7%)	0.021
Intact cord during stimulation			
No, 1892	1303(70.5%)	589(82.3%)	
Yes, 671	544(29.5%)	127(17.7%)	<0.0001
Drying after birth			
No, 159	100(5.4%)	59(8.2%)	
Yes, 2404	1747(94.6%)	657(91.8%)	0.01
Gestational age in weeks (SD)	38.53±2.0	38.2±2.7	
Preterm birth (<37 weeks)			
No, 2272	1657 (89.7%)	615 (85.9%)	
Yes, 291	190 (10.3%)	101 (14.1%)	0.008
Birth weights in gram (SD)	2836.9±523.9	2707.6±614.0	
Low Birth Weight (<2500 gram)			
No, 2083	1543 (83.8%)	540 (76.6%)	
Yes, 463	298 (16.2%)	165 (23.4%)	<0.0001

Confidential: For Review Only

Table 4. Bi-variate and multi-variate analysis of association between obstetrics and neonatal characteristics with breathing after stimulation

	Bi-variate analysis			Multi-variate analysis		
	Beta coefficient	p-value	cOR (95% CI)	Beta coefficient	p-value	aOR (95% CI)
Global intercept				0.225		1.252
intact cord (reference cord clamped)						
Intercept	0.133		1.143			
Intact cord	0.661	<0.0001	1.94 (1.56, 2.40)	0.609	<0.0001	1.84 (1.48, 2.29)
Complication during admission						
Intercept	1.034		2.814			
Yes	-0.701	<0.0001	0.50 (0.38, 0.64)	-0.676	<0.0001	0.51 (0.39, 0.66)
Mode of delivery (reference spontaneous birth)						
Intercept	1.304	0	3.683			
Instrumental delivery	-0.313	0.019	0.73 (0.56, 0.95)	-0.283	0.037	0.75 (0.58, 0.98)
Immediate drying after birth (reference no drying)						
Intercept	0.528	0.001	1.695			
Immediate drying after birth	0.45	0.008	1.57 (1.12, 2.19)	0.4	0.022	1.49 (1.06, 2.10)
Low Birth Weight (reference 2500 gram or more)						
Intercept	1.05	0	2.857			
Low Birth Weight	-0.459	0	0.63 (0.51, 0.78)	-0.383	0.003	0.68 (0.53, 0.88)
Preterm (reference 37 weeks or more)						
Constant	0.991	0	2.694			
Preterm1	-0.359	0.006	0.70 (0.54, 0.90)	0.041	0.8	1.04 (0.76, 1.43)

Confidential: For Review Only

Figure 1. Study flow figure

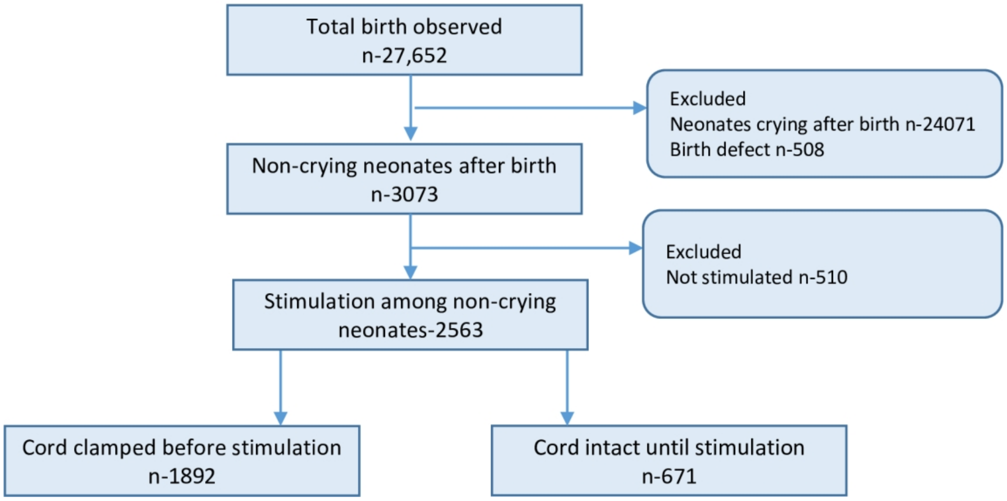

150x86mm (300 x 300 DPI)

Figure 2. Breathing after stimulation among non-crying babies with or without intact cord

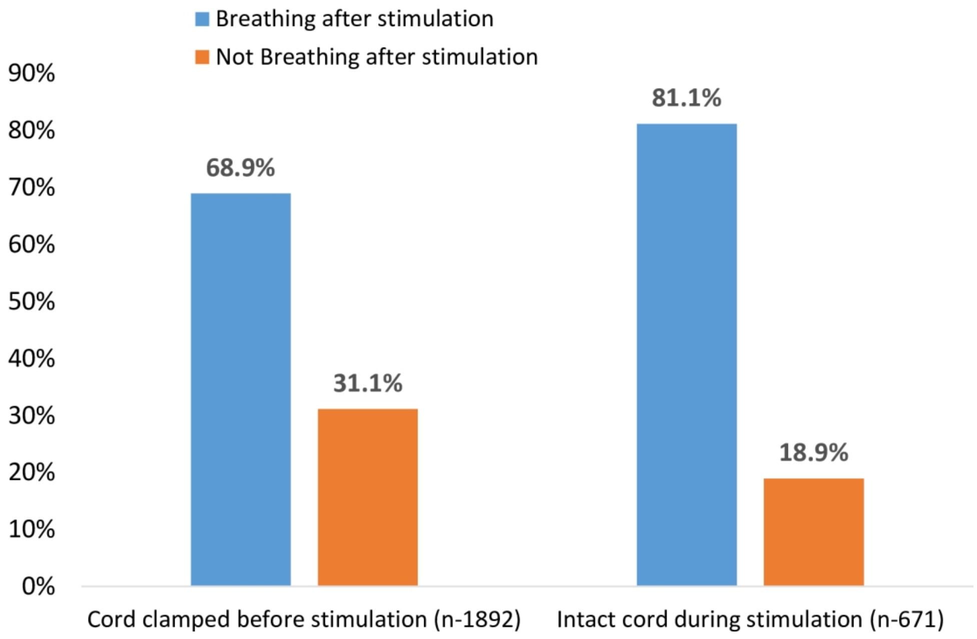

152x108mm (300 x 300 DPI)

Impact of stimulation among non-crying neonates with intact cord versus clamped cord on birth outcomes- observation study

Ashish KC[1], Shyam Sundar Budathoki[2,3], Jeevan Thapa[4], Susan Niermeyer[5], Rejina Gurung[1,3], Nalini Singhal[6] on behalf of Nepal Neonatal Network[ø]

[1]Department of Women's and Children's Health, Uppsala University, Uppsala, Sweden;

[2]Department of Primary Care and Public Health, School of Public Health, Imperial College London, London, United Kingdom;

[3]Golden Community, Lalitpur, Nepal;

[4] Department of Community Health Sciences, Patan Academy of Health Sciences, Lalitpur, Nepal;

[5]University of Colorado School of Medicine, Colorado School of Public Health, Aurora, CO, USA;

[6]Department of Pediatrics, University of Calgary, Calgary, Canada.

Correspondence- Ashish KC, Department of Women's and Children's Health, Uppsala University, SE-751 85, Uppsala, Sweden, www.kbh.uu.se/imch. Email address: ashish.k.c@kbh.uu.se; Phone number: +46 0729001074

Confidential: For Review Only

ABSTRACT

Background

Stimulation of non-crying neonates after birth can help transition to spontaneous breathing. In this study, we aim to assess the impact of intact versus clamped umbilical cord on spontaneous breathing after stimulation of non-crying neonates.

Methods

This is an observational study among non-crying neonates (n=3,073) born in hospitals of Nepal. Non-crying neonates born vaginally at gestational age ≥34-weeks were observed for their response to stimulation with the cord intact or clamped. Obstetric characteristics of the neonates were analysed. Association of spontaneous breathing with cord management was assessed using logistic regression.

Results

Among non-crying neonates, 2,563 of them received stimulation. Of these, a higher proportion of the neonates were breathing in the cord intact group compared to those cord clamped group (81.1% vs 68.9%, p<0.0001). The use of bag-and-mask ventilation was lower among those who were stimulated with the cord intact than those who were stimulated with cord clamped (18.0% vs 32.4%, p<0.0001). The proportion of neonates with Apgar-score ≤3 at 1 minute was lower with the cord intact than with cord clamped (7.6% vs 11.5%, p-0.001). In multi-variate analysis, neonates with intact cord had 84%

Confidential: For Review Only

increased odds of spontaneous breathing (aOR, 1.84; 1.48, 2.29) compared to those with cord clamped.

Conclusions

Stimulation of non-crying neonates with intact cord was associated with more spontaneous breathing than who were stimulated with cord clamped. This study provides evidence on the effect of the intact cord during stimulation. There are potential benefits to performing the initial steps of resuscitation with the cord intact.

Stimulation of non-crying neonates with intact cord was associated with more spontaneous breathing than who were stimulated with cord clamped. This indicates that primary apnoeic neonate transition better when stimulated with cord intact.

Key words: non-crying neonates, stimulation with cord intact, spontaneous breathing, resuscitation.

Confidential: For Review Only

What is already known on this topic?

- Stimulation is one of the key steps to neonatal resuscitation
- Breathing prior to umbilical cord clamping has been shown to result in smoother cardiovascular transition at birth in experimental studies

What this study adds?

- Stimulation with the cord intact may be more effective in triggering spontaneous breathing and result in better Apgar scores than stimulation provided after cord clamping.

Confidential: For Review Only

INTRODUCTION

Globally, every year among the 140 million neonates who are born, 10-15 million do not cry or breathe at birth[1]. These babies require resuscitation to transition from the intrauterine to the extra-uterine environment[2]. Neonates who do not receive timely and adequate resuscitation may die or suffer brain injuries and long-term disability[3]. Every year, an estimated 1 million neonates die due to intrapartum related complications or "birth asphyxia"[4], 2 million experience hypoxic-ischemic encephalopathy and 1.2 million go on to show developmental delay[3]. Most of this mortality and morbidity occur in low- and middle-income countries.

Transitioning to an extra-uterine environment depends on two major physiological events, the commencement of breathing and the transition of blood flow from the umbilical to the pulmonary circulation[2 5]. The trigger of breathing at birth results in absorption of the liquid in the trachea and airways, aeration of the lungs, reduction in pulmonary vascular resistance, and increase in pulmonary blood flow[6 7]. This increase in pulmonary blood flow supplies left ventricular preload previously dependent on flow across the patent foramen ovale from the right atrium[2]. Any intrapartum insult due to placental insufficiency (hypertension, infection or haemorrhage) can delay the spontaneous cardiopulmonary transition and may be compounded by umbilical cord clamping [8 9]. As a result, blood flows continuously through the ductus arteriosus, by-passing the pulmonary circulation into the systemic circulation as right to left shunting[6]. Thus, resuscitation with the cord intact has been recommended.

Confidential: For Review Only

The International Liaison Committee on Resuscitation provides guidance on neonatal resuscitation[10]. The ILCOR 2020 recommends that a neonate who is not crying or breathing with poor tone and heart rate less than 100 bpm should receive stimulation and clearing of airways (as needed) with intact cord[10]. There is very little robust evidence on the impact of stimulation in neonates receiving resuscitation with the cord intact. The World Health Organization (WHO) recommends if there is an experience in providing ventilation without cutting the umbilical cord, ventilation can be initiated before cord-cutting.[11] WHO identifies resuscitation with the cord intact as a key research area to generate a stronger evidence base for care.[11].

Studies from low and middle-income countries have reported that neonates who are not crying or breathing at birth are immediately taken to a neonatal resuscitation area for stimulation, clearing of airways and positive-pressure ventilation[12 13]. Despite educational programs such as Helping Babies Breathe (HBB) from the American Academy of Pediatrics, which advocates stimulation to breathe before clamping the cord, health workers often clamp the cord before initiating resuscitation [14].

This study aims to evaluate the impact of stimulation among neonates with the cord intact versus those with cord clamped on breathing and birth outcome.

METHODS:

Study Design: This was a nested observational study within two quality improvement studies SUSTAIN[15] and REFINE[16] done in nine hospitals of Nepal.

Confidential: For Review Only

Study Sites: This study was conducted among nine hospitals in Nepal. The hospitals are tertiary care hospitals providing referral obstetric services through Comprehensive and Emergency Obstetric and Neonatal Care Services. These hospitals were chosen for those quality improvement studies to implement a safer birth bundle package. Health workers were trained on HBB 2.0 to resuscitate neonates with intact cord where possible as part of the safer birth bundle. The cord management was at the discretion of the individual health worker.

Study Dates: The study was conducted between 1 June 2019 to 2 May 2020.

Participants: Women in labour with gestational age ≥34 >33 weeks and confirmed fetal heart rate during admission were considered eligible for the study and approached for consent. Those women who underwent caesarean section for delivery, or whose birth were not observed were excluded from the study. Non-crying newborns who received additional tactile stimulation were included in the study. Furthermore, the babies who cried immediately after birth and those with birth defects were excluded from the study. Neonates who did not cry were included in the analysis.

Sample size: All non-crying neonates who received tactile stimulation were enrolled in the large study were considered for analysis.

Variables: The variables considered were defined as cord status: intact and clamped; Parity: no previous birth, 1 previous birth and 2 or more previous births; maternal complication during admission: any medical/obstetric complications during admission of the women for labour, induction of labour, augmentation of labour; mode of

Confidential: For Review Only

delivery: instrumental birth (for forceps, vacuum application) or spontaneous; preterm birth (age of gestation less than 37 weeks); low birth weight (weight of baby less than 2500 gm); immediate thorough drying after birth and breathing after stimulation (spontaneous breathing requiring no positive pressure ventilation). Stillbirth defined as no heart rate or breathing after resuscitation action.

Data collection and Management: The data collection was done through the existing data collection systems of the two studies REFINE and SUSTAIN. Each of the participating hospitals had systems for individual-level data collection. Observational data was collected by independent clinical researchers using a tablet-based application. They also collected obstetric and neonatal information for the patient case notes. They recorded information on induction, augmentation of labour, complications during admission (antepartum haemorrhage, hypertensive disorder, premature rupture of member, abnormal presentation, infection), mode of delivery (spontaneous and instrumental vaginal), sex and weight of baby, crying status of baby, and whether drying and clearing of the airway were performed. An independent database manager reviewed the data entered in the tablet-based application weekly. The data was then extracted into SPSS software for cleaning, coding and analysis.

Data Analysis: The non-crying babies included in the study were categorised as those who were stimulated with intact cord and those who were stimulated after cord

Confidential: For Review Only

clamping. The background characteristics among these two groups were compared. A comparison was done for obstetric and neonatal characteristics.

Spontaneous bBreathing after stimulation was observed among these two groups to see the impact of intact versus clamped cord at the time of stimulation. The multivariable analysis using logistic regression was done to assess the association of cord status during stimulation on breathing by adjusting for the variables which had a significant level difference of p<=0.01. The variables which were significantly different in cord status during stimulation were complications during admission, mode of delivery and immediate drying.

Confidential: For Review Only

Ethical Approval: Ethical approval was granted by the Ethical Review Board of the Nepal Health Research Council for SUSTAIN (ref. 110/2019) and REFINE studies (ref. 87/2018). Written informed consent was obtained from the participants at the time of admission to the hospital before enrolling in the study.

RESULTS:

During the study period, 41,621 women delivered in the hospitals and 34,652 women consented to the study. Among the consented women 27,652 births were observed in the study. Among the total births observed 24,071 babies were excluded as they cried immediately after birth, and 508 births were further excluded due to birth defects; 3073 non-crying neonates were included in the analysis. Among the non-crying neonates, 2563 were stimulated. Of the neonates stimulated, 671 (26.2%) of them had their cord intact while 1892 (73.8%) had their cord clamped (figure 1). Of the neonates who had

the cord intact during stimulation, 81.1% of them had smooth and spontaneous breathing, while 68.9% of the neonates with cord clamped were breathing after stimulation (p-value <0.0001) (figure 2).

We compared the obstetric and neonatal characteristics of neonates who had cord intact versus cord clamped before stimulation. Maternal complication during admission was lower among neonates who had cord intact than those who had the cord clamped Among the neonates whose mother had complications during admission, intact cord during stimulation was lower than if mother had no complication (8.0% vs 1120.26% vs 26.9%, p-value=0.022). For neonates delivered afterThe induction of labour was lower among neonates who had their, the proportion with the cord intact than those who had the cord clamped was lower than among neonates delivered after spontaneous labor (18.5% vs 250.5% vs 28.0%, p-value <0.0001). Among neonates who were born through instrumental delivery, the proportion with cord intact was lower than those who had the cord clamped among those born by spontaneous vaginal route (819.4% vs 12.37.8%, p-value=0.005) (Table 1).

The clinical outcome differed among neonates stimulated with the cord intact vs with those cord clamped. Among neonates stimulated with the cord intact, 81.1% breathed, while 68.9% breathed after stimulation with cord clamped (p-value <0.0001). The use of bag-and-mask ventilation was lower among those who were stimulated with the cord intact than those who were stimulated with cord clamped (18.0% vs 32.4%, p-

Confidential: For Review Only

value <0.0001). The proportion of neonates with Apgar score 3 or less at 1 minute was higher~~lower~~ with the cord intact~~tt~~ than those who were cord clamped (92.1~~7.6~~% vs 88.3~~11.5~~%, p-value 0.001). The proportion of neonates with Apgar score 6~~5~~ or less at 5 minutes was higher~~lower~~ with the cord intact than those who were cord clamped (~~7.~~92.1% vs 8~~12.~~7.3% p-value <0.0001). The proportion of mortality was lower among the neonates who had their cord intact but statistically not significantly different from those with cord clamped (0.7 vs 1.6%, p-value 0.123) (Table 2).

We compared the obstetric and neonatal characteristics of non-crying neonates who breathed after stimulation. 58.3% of neonates with a maternal complication during admission breathed after stimulation, while 73.8% of neonates without maternal complication breathed after stimulation (p-value <0.0001). 66.3% of neonates who were born through instrumental delivery breathed after stimulation, while 72.9% of neonates who were born through spontaneous delivery breathed after stimulation (p-value=0.021). 62.9% of neonates who were not immediately dried after birth breathed after stimulation, while 72.7% of neonates who were immediately dried after birth breathed after additional stimulation (p-value=0.01). (Table 3)

In bivariate analysis, there is 1.94~~%~~ higher odds of breathing if the cord was intact during stimulation compared to the neonates with cord clamped (cOR, 1.94; 95% CI; 1.56, 2.40). If the neonate's mother had complications during admission for delivery, the odds of breathing after stimulation was 0.50~~%~~ lower than neonate's whose mother had no complication during admission (cOR, 0.50; 95% CI; 0.38, 0.64). Neonates who

Confidential: For Review Only

were dried immediately after birth had 1.57% higher odds of breathing after stimulation than those who were not dried after birth (cOR, 1.57; 95% CI; 1.12, 2.19).. In neonates with birth weight 2500 grams, the odds of breathing after stimulation was 0.63 lower than neonate's with low birth weight (cOR, 0.63; 95% CI; 0.51, 0.78). (Table 4)

In multi-variate analysis, to assess the association of characteristics with breathing after stimulation, there was 1.84% higher odds of breathing if the cord was intact during stimulation when compared to neonates with cord clamped (aOR, 1.84; 95% CI; 1.48, 2.29) after adjusting with a maternal complication during admission, mode of delivery, and immediate drying after birth, low birth weight and preterm birth. Neonates who were dried immediately after birth had 1.49% higher odds of breathing after stimulation than those who were not dried after birth (aOR, 1.49; 95% CI; 1.06, 2.10). (Table 4)

DISCUSSION

In this observational study, one-fourth of the non-crying neonates had stimulation performed with an intact cord. There was higher odds of 84% for spontaneous breathing if the cord was kept intact during stimulation than neonates who had their cord clamped during stimulation. The higher maternal complication during admission and apgar score less than 4 at 1 minute among early cord clamped non-crying neonates indicates that these are more depressed neonates than neonates with cord intact. This was explained by higher proportion of bag-and-mask ventilation among

Confidential: For Review Only

early cord clamped neonates. The odds of breathing after stimulation increased if neonates were immediately dried after birth and their cord remained intact during stimulation. The proportion of neonates who required bag-and-mask ventilation decreased by half if neonates were stimulated with the cord intact. Even though there was no significant difference in mortality between the two groups, the mortality trended lower among the neonates who were stimulated with the cord intact.

Similar to our results, a single-center randomized controlled trial (RCT) in California, US to evaluate the effect of delayed cord clamping in premature infants (<32 weeks) who required ventilation demonstrated that provision of gentle tactile stimulation with the cord intact may hasten the establishment of spontaneous respirations and provide a similar placental transfusion compared to positive-pressure ventilation with delayed cord clamping.[17] Another multi-centric trial in the UK among premature infants (≤ 32 weeks) suggests that cord clamping after at least 2 min and providing neonatal care with the cord intact may improve outcome at discharge for the infants compared with early clamping and neonatal care after clamping.[18]

A single-center RCT in Nepal evaluated the effect on delayed versus cord clamping in babies requiring resuscitation[19]. In the trial, randomization was performed while the baby was still in utero, and resuscitation measures (stimulation and positive pressure ventilation) performed according to the HBB algorithm with an unclamped cord in the mother's bed were associated with early spontaneous breathing.

https://mc.manuscriptcentral.com/bmjpo

In an experimental animal study by Bhatt et al. demonstrated a more stable cardiovascular adaptation if cord clamping was performed after initiation of ventilation[7]. The results from this and other studies suggested that resuscitation of newborns should be performed with an uncut cord, facilitating the postnatal transition[20-22]. However, in a single-center observation study in Tanzania, there was no significant association between time to cord clamping and onset of breathing or initiation of positive-pressure ventilation following stimulation/suction[23].

Our multi-centric study conducted between 2017-2018 showed that 9.8% of neonates do not cry at birth[12]. The study found that non-crying but breathing babies might not have the full respiratory capacity and as a result required resuscitation.[24] In this study, we hypothesize that the non-crying neonates who breathe spontaneously after stimulation might have been in primary apnea. There is an increased oxygenation with intact cord due to several interacting mechanisms; persisting oxygenation by the placenta, avoidance of bradycardia often associated with clamping the cord before the onset of respirations, and earlier initiation of breathing resulting in increased pulmonary blood flow.

A study in Tanzania showed that although the health care provider commonly practiced clamping the umbilical cord immediately after delivery, they were aware that delayed cord clamping has a potential benefit of oxygenation to the newborn in the event of the need for resuscitation.[25] Our study also shows that despite the education of health care providers on keeping the cord intact during resuscitation, two-thirds of

Confidential: For Review Only

the non-crying babies received resuscitation after cord clamping. There is a need to implement a delay in clamping as a quality improvement intervention to improve initial steps of resuscitation especially stimulation and if possible bag-and-mask ventilation. Three recent systematic reviews and meta-analysis showed that there is a lack of evidence to recommend cord management among term and preterm infants who receive positive-pressure ventilation at birth.[26 27]

Methodological consideration

This study has several strengths including that observations were done by trained researchers in multiple hospitals in Nepal in a consistent way and with large sample size. Using observational methods limited recall bias. However, there are several limitations. First, this observation was only done for vaginal births and not for caesarean births. Second, the discretion of cord management was decided by health workers, so there is a bias toward the pre-existing cord clamping practice despite being trained on keeping the cord intact during stimulation. Finally, we did not have cord gas values to determine if babies who did not cry had perinatal acidosis or asphyxia.

In conclusion, our study found offer non-crying neonates stimulated with the cord intact more like to breathe than those who are stimulated with cord clamped. This approach will provide the added advantage that stimulation provided by a single provider can induce spontaneous respiration with the cord intact in primary apneic neonates and would not require ventilation and additional providers, especially in low-resource settings. Our study provides additional evidence consistent with the ILCOR

Confidential: For Review Only

recommendation on keeping the cord intact during the initial steps of resuscitation. Despite the training on updated neonatal resuscitation to keep the cord intact, still, three-fourths of the neonates who are not crying have their cord clamped. Further research, education and quality improvement interventions are required to improve cord management among neonates who require help to breathe at birth.

ᴓNepal Neonatal Network-Omkar Basnet, Srijana Sharma, Pratikshya Bhattarai, Ankit Acharya, Prof. Anjani Kumar Jha, Dr. Yasho Vardhan Pradhan, Dr. Sushil Nath Pyakurel, Prof. Kiran Bajracharya, Dr. Sheela Verma, Dr. Amit Bhandari, Dr. Anjana KC Thapa, Dr. Krishna Prasad Bista, Jhalak Sharma Paudel, Sangita Khatri, and Leela Paudel.

Author's contribution-AKC and NS conceptualized the study. AKC, JT and SSB conducted the analysis. NS, RG provided feedback to the analysis. AKC, SSB, RG and JT wrote the first draft. NS and SN made the second and final revision to the draft. All authors reviewed and provided feedback on the revised version and agreed to the final version.

Conflict of Interest-We declare no conflict of interest

Consent for Publication-None

Availability of supporting data

The dataset generated and analysed is not publicly available as it is part of larger quality improvement projects but can be made available on reasonable request with a data-sharing agreement.

Confidential: For Review Only

Competing interests-The authors declare that they have no competing interests.

Funding-No specific funding was available for this study.

Acknowledgement-We would like to thank all the data coordinators and surveillance officers, hospital manager, nursing in-charge, all the nurses and support staff working in the sick newborn care units. We thank all the mothers and caregivers of the sick newborns who consented to the study.

Confidential: For Review Only

References

1. Lee AC, Cousens S, Wall SN, et al. Neonatal resuscitation and immediate newborn assessment and stimulation for the prevention of neonatal deaths: a systematic review, meta-analysis and Delphi estimation of mortality effect. *BMC Public Health* 2011;11 Suppl 3:S12. doi: 10.1186/1471-2458-11-S3-S12 [published Online First: 2011/04/29]

2. Hooper SB, Polglase GR, te Pas AB. A physiological approach to the timing of umbilical cord clamping at birth. *Arch Dis Child Fetal Neonatal Ed* 2015;100(4):F355-60. doi: 10.1136/archdischild-2013-305703 [published Online First: 2014/12/30]

3. Lee AC, Kozuki N, Blencowe H, et al. Intrapartum-related neonatal encephalopathy incidence and impairment at regional and global levels for 2010 with trends from 1990. *Pediatr Res* 2013;74 Suppl 1:50-72. doi: 10.1038/pr.2013.206 [published Online First: 2013/12/25]

4. Liu L, Oza S, Hogan D, et al. Global, regional, and national causes of under-5 mortality in 2000-15: an updated systematic analysis with implications for the Sustainable Development Goals. *Lancet* 2016;388(10063):3027-35. doi: 10.1016/S0140-6736(16)31593-8 [published Online First: 2016/11/15]

5. Gao Y, Raj JU. Regulation of the pulmonary circulation in the fetus and newborn. *Physiol Rev* 2010;90(4):1291-335. doi: 10.1152/physrev.00032.2009 [published Online First: 2010/10/21]

6. Crossley KJ, Allison BJ, Polglase GR, et al. Dynamic changes in the direction of blood flow through the ductus arteriosus at birth. *J Physiol* 2009;587(Pt 19):4695-704. doi: 10.1113/jphysiol.2009.174870 [published Online First: 2009/08/14]

7. Bhatt S, Alison BJ, Wallace EM, et al. Delaying cord clamping until ventilation onset improves cardiovascular function at birth in preterm lambs. *J Physiol* 2013;591(8):2113-26. doi: 10.1113/jphysiol.2012.250084 [published Online First: 2013/02/13]

8. Polglase GR, Dawson JA, Kluckow M, et al. Ventilation onset prior to umbilical cord clamping (physiological-based cord clamping) improves systemic and cerebral oxygenation in preterm lambs. *PLoS One* 2015;10(2):e0117504. doi: 10.1371/journal.pone.0117504 [published Online First: 2015/02/18]

9. Katheria AC, Brown MK, Rich W, et al. Providing a Placental Transfusion in Newborns Who Need Resuscitation. *Front Pediatr* 2017;5:1. doi: 10.3389/fped.2017.00001 [published Online First: 2017/02/10]

10. Aziz K, Lee CHC, Escobedo MB, et al. Part 5: Neonatal Resuscitation 2020 American Heart Association Guidelines for Cardiopulmonary Resuscitation and Emergency Cardiovascular Care. *Pediatrics* 2021;147(Suppl 1) doi: 10.1542/peds.2020-038505E [published Online First: 2020/10/23]

11. Organization WH. Guidelines on basic newborn resuscitation. In: WHO, ed. Geneva: WHO, 2012.

12. Kc A, Lawn JE, Zhou H, et al. Not Crying After Birth as a Predictor of Not Breathing. *Pediatrics* 2020;145(6) doi: 10.1542/peds.2019-2719 [published Online First: 2020/05/14]

13. Kc A, Ewald U, Basnet O, et al. Effect of a scaled-up neonatal resuscitation quality improvement package on intrapartum-related mortality in Nepal: A stepped-wedge cluster randomized controlled trial. *PLoS Med* 2019;16(9):e1002900. doi: 10.1371/journal.pmed.1002900 [published Online First: 2019/09/10]

14. Kamath-Rayne BD, Thukral A, Visick MK, et al. Helping Babies Breathe, Second Edition: A Model for Strengthening Educational Programs to Increase Global Newborn Survival. *Glob Health Sci Pract* 2018;6(3):538-51. doi: 10.9745/GHSP-D-18-00147 [published Online First: 2018/10/06]

15. Gurung R, Jha AK, Pyakurel S, et al. Scaling Up Safer Birth Bundle Through Quality Improvement in Nepal (SUSTAIN)-a stepped wedge cluster randomized controlled trial in public hospitals. *Implement Sci* 2019;14(1):65. doi: 10.1186/s13012-019-0917-z [published Online First: 2019/06/21]

Confidential: For Review Only

16. Gurung R, Gurung A, Basnet O, et al. REFINE (Rapid Feedback for quality Improvement in Neonatal rEsuscitation): an observational study of neonatal resuscitation training and practice in a tertiary hospital in Nepal. *BMC Pregnancy Childbirth* 2020;20(1):756. doi: 10.1186/s12884-020-03456-z [published Online First: 2020/12/05]
17. Katheria A, Poeltler D, Durham J, et al. Neonatal Resuscitation with an Intact Cord: A Randomized Clinical Trial. *J Pediatr* 2016;178:75-80 e3. doi: 10.1016/j.jpeds.2016.07.053 [published Online First: 2016/10/30]
18. Duley L, Dorling J, Pushpa-Rajah A, et al. Randomised trial of cord clamping and initial stabilisation at very preterm birth. *Arch Dis Child Fetal Neonatal Ed* 2018;103(1):F6-F14. doi: 10.1136/archdischild-2016-312567 [published Online First: 2017/09/20]
19. Andersson O, Rana N, Ewald U, et al. Intact cord resuscitation versus early cord clamping in the treatment of depressed newborn infants during the first 10 minutes of birth (Nepcord III) - a randomized clinical trial. *Matern Health Neonatol Perinatol* 2019;5:15. doi: 10.1186/s40748-019-0110-z [published Online First: 2019/09/06]
20. Niermeyer S, Velaphi S. Promoting physiologic transition at birth: re-examining resuscitation and the timing of cord clamping. *Semin Fetal Neonatal Med* 2013;18(6):385-92. doi: 10.1016/j.siny.2013.08.008 [published Online First: 2013/09/24]
21. Mercer JS, Erickson-Owens DA. Is it time to rethink cord management when resuscitation is needed? *J Midwifery Womens Health* 2014;59(6):635-44. doi: 10.1111/jmwh.12206 [published Online First: 2014/10/10]
22. Bjorland PA, Ersdal HL, Eilevstjonn J, et al. Changes in heart rate from 5 s to 5 min after birth in vaginally delivered term newborns with delayed cord clamping. *Arch Dis Child Fetal Neonatal Ed* 2020 doi: 10.1136/archdischild-2020-320179 [published Online First: 2020/11/12]
23. Ersdal HL, Linde J, Auestad B, et al. Timing of cord clamping in relation to start of breathing or ventilation among depressed neonates-an observational study. *BJOG* 2016;123(8):1370-7. doi: 10.1111/1471-0528.13778 [published Online First: 2015/12/25]
24. David G Tingay, Olivia Farrell, Jessica Thomson, et al. Imaging the Respiratory Transition at Birth: Unravelling the Complexities of the First Breaths of Life. *American Journal of Respiratory and Critical Care Medicine* 2021 February (accepted)
25. Mwakawanga DL, Mselle LT. Early or delayed umbilical cord clamping? Experiences and perceptions of nurse-midwives and obstetricians at a regional referral hospital in Tanzania. *PLoS One* 2020;15(6):e0234854. doi: 10.1371/journal.pone.0234854 [published Online First: 2020/06/23]
26. Jeevan A, Ananthan A, Bhuwan M, et al. Umbilical cord milking versus delayed cord clamping in term and late-preterm infants: a systematic review and meta-analysis. *J Matern Fetal Neonatal Med* 2021:1-11. doi: 10.1080/14767058.2021.1884676 [published Online First: 2021/02/12]
27. Seidler AL, Gyte GML, Rabe H, et al. Umbilical Cord Management for Newborns <34 Weeks' Gestation: A Meta-analysis. *Pediatrics* 2021;147(3) doi: 10.1542/peds.2020-0576 [published Online First: 2021/02/27]

Confidential: For Review Only

Tables and figures

Table 1. Obstetric and neonatal characteristics among non-crying neonates with intact cord versus cord clamped during stimulation

-	Intact cord during stimulation n-671	Cord clamped before stimulation n-1892	p-value
Median time to cord clamping (IQR) seconds	58.0 (34, 71)	25 (18, 31)	
Parity			
Nullipara, 1430	376 (56.5%)	1054 (56.0%)	0.892
Primipara, 714	180 (27.0%)	534 (28.4%)	0.515
Multipara, 403	110 (16.5%)	293 (15.6%)	0.579
Complication during admission			
No, 2292	617(92.0%)	1675(88.8%)	
Yes, 266	54 (8.0%)	212 (11.2%)	0.022
Induction of labour			
No, 1957	547 (81.5%)	1410(74.5%)	
Yes, 606	124(18.5%)	482(25.5%)	<0.0001
Augmentation of labour			
No, 1600	445(66.3%)	1155(61.0%)	
Yes, 963	226(33.7%)	737(39.0%)	0.016
Mode of delivery			
Spontaneous, 2260	611(91.6%)	1649(87.7%)	
instrumental, 288	56(8.4%)	232(12.3%)	0.005
Gestational age in weeks (SD)	38.4±2.2	38.5±2.3	
Preterm (<37 weeks)			
No, 2272	594(88.5%)	1678(88.7%)	
Yes, 291	77(11.5%)	214(11.3%)	0.944
Birth weight in weeks (SD)	2784.3±543.7	2807.1±556.6	
Low birth weight (<2500 gram)			
No, 2083	537(82.3%)	1546(82.3%)	
Yes, 463	131(19.6%)	332(17.7%)	0.268
Immediate drying			
No, 159	35(5.2%)	124(6.6%)	
Yes, 2404	636(94.8%)	1892(93.4%)	0.227
			p-value
-			-
Parity			-
No previous birth			0.892
1 previous birth			0.515
2 or more previous birth			0.579

Confidential: For Review Only

~~Complication during admission~~		-
~~No~~		-
~~Yes~~		~~0.022~~
~~Induction of labour~~		-
~~No~~		-
~~Yes~~		~~<0.0001~~
~~Augmentation of labour~~		-
~~No~~		-
~~Yes~~		~~0.016~~
~~Mode of delivery~~		-
~~Spontaneous~~		-
~~instrumental~~		~~0.005~~
~~Low birth weight babies~~		-
~~No~~		-
~~Yes~~		~~0.268~~
~~Preterm birth~~		-
~~No~~		-
~~Yes~~		~~0.944~~
~~Immediate drying after birth~~		-
~~No~~		-
~~Yes~~		~~0.227~~

Confidential: For Review Only

Confidential: For Review Only

Table 2. Clinical outcome of non-crying neonates who were stimulated with cord intact vs cord clamped

-	Intact cord during stimulation n-671 (26.2%)	Cord clamped before stimulation n-1892 (73.8%)	p-value
Spontaneous breathing			
Yes, 1847	544 (81.1%)	1303 (68.9%)	
No, 716	127 (18.9%)	589 (31.1%)	<0.0001
Bag-and-mask ventilation			
No, 1829	550 (82.0%)	1279 (67.6%)	
Yes, 734	121 (18.0%)	613 (32.4%)	<0.0001
Apgar score at 1 minute			
≤4, 2214	598 (92.4%)	1616 (88.3%)	
≥3, 264	49 (7.6%)	215 (11.5%)	0.001
Apgar score at 5 minute			
≤7, 2196	596 (92.1%)	1597 (87.3%)	
>6, 284	51 (7.9%)	233 (12.7%)	<0.0001
Birth outcome			
Live born, 2513	654 (97.5%)	1859 (98.3%)	
Stillborn, 50	17 (2.5%)	33 (1.7%)	0.198
Mortality			
No, 2528	666 (99.3%)	1862 (98.4%)	
Yes, 35	5 (0.7%)	30 (1.6%)	0.123
-		~~Cord clamped before stimulation n-1892 (73.8%)~~	~~p-value~~
~~Breathing~~		-	-
~~Yes~~		~~1303 (68.9%)~~	-
~~No~~		~~589 (31.1%)~~	~~<0.0001~~
~~Bag-and-mask ventilation~~		-	-
~~No~~		~~1279 (67.6%)~~	-
~~Yes~~		~~613 (32.4%)~~	~~<0.0001~~
~~Apgar score at 1 minute~~		-	-
~~≤4~~		~~1616 (88.3%)~~	-
~~≥3~~		~~215 (11.5%)~~	~~0.001~~
~~Apgar score at 5 minute~~		-	-
~~≤7~~		~~1597 (87.3%)~~	-
~~>6~~		~~233 (12.7%)~~	~~<0.0001~~

Confidential: For Review Only

Birth outcome	-	-
Live born	1859 (98.3%)	-
Stillborn	33 (1.7%)	0.198
Mortality	-	-
No	1862 (98.4%)	-
Yes	30 (1.6%)	0.123

Confidential: For Review Only

Table 3. The obstetric and neonatal characteristics of non-crying neonates who responded to stimulation

~~-~~	~~Breathing after stimulation (n=1847)~~	~~Not breathing after stimulation (n=716)~~	~~p-value~~
~~-~~	~~72.1 (70.3, 73.8)~~	~~27.9 (26.2, 29.7)~~	~~-~~
~~Complication during admission~~	~~-~~	~~-~~	~~-~~
~~No~~	~~73.8% (1691)~~	~~26.2% (601)~~	~~-~~
~~Yes~~	~~58.3% (155)~~	~~41.7% (111)~~	~~<0.0001~~
~~Induction of labour~~	~~-~~	~~-~~	~~-~~
~~No~~	~~71.8% (1406)~~	~~28.2% (551)~~	~~-~~
~~Yes~~	~~72.8% (441)~~	~~27.2% (165)~~	~~0.679~~
~~Augmentation of labour~~	~~-~~	~~-~~	~~-~~
~~No~~	~~72.4% (1159)~~	~~27.6% (441)~~	~~-~~
~~Yes~~	~~71.4% (688)~~	~~28.6% (275)~~	~~0.586~~
~~Mode of delivery~~	~~-~~	~~-~~	~~-~~
~~Spontaneous~~	~~72.9% (1648)~~	~~27.1% (612)~~	~~-~~
~~Instrumental~~	~~66.3% (191)~~	~~33.7% (97)~~	~~0.021~~
~~Intact cord during stimulation~~	~~-~~	~~-~~	~~-~~
~~No~~	~~68.9% (1303)~~	~~31.1% (589)~~	~~-~~
~~Yes~~	~~81.1% (544)~~	~~18.9% (127)~~	~~<0.0001~~
~~Immediate drying after birth~~	~~-~~	~~-~~	~~-~~
~~No~~	~~62.9% (100)~~	~~37.1% (59)~~	~~-~~
~~Yes~~	~~72.7% (1747)~~	~~27.3% (657)~~	~~0.01~~

	Breathing after stimulation (n=1847	Not breathing after stimulation (n=716)	p-value
Complication during admission			
No, 2292	1691(91.6%)	601(84.4%)	
Yes, 266	155(8.4%)	111(15.6%)	<0.0001
Induction of labour			
No, 1957	1406(76.1%)	551(77.0%)	
Yes, 606	441(23.9%)	165(23.0%)	0.679
Augmentation of labour			
No, 1600	1159(62.8%)	441(61.6%)	
Yes, 963	688(37.2%)	275(38.4%)	0.586
Mode of delivery			
Spontaneous, 2260	1648(89.6%)	612(86.3%)	
Instrumental, 288	191(10.4%)	97(13.7%)	0.021
Intact cord during stimulation			
No, 1892	1303(70.5%)	589(82.3%)	
Yes, 671	544(29.5%)	127(17.7%)	<0.0001

Confidential: For Review Only

Drying after birth	_	_	_
No, 159	100(5.4%)	59(8.2%)	_
Yes, 2404	1747(94.6%)	657(91.8%)	0.01
Gestational age in weeks (SD)	38.53±2.0	38.2±2.7	_
Preterm birth (<37 weeks)	_	_	_
No, 2272	1657 (89.7%)	615 (85.9%)	_
Yes, 291	190 (10.3%)	101 (14.1%)	0.008
Birth weights in gram (SD)	2836.9±523.9	2707.6±614.0	_
Low Birth Weight (<2500 gram)	_	_	_
No, 2083	1543 (83.8%)	540 (76.6%)	_
Yes, 463	298 (16.2%)	165 (23.4%)	<0.0001

Confidential: For Review Only

Table 4. Bi-variate and multi-variate analysis of association between obstetrics and neonatal characteristics with breathing after stimulation

	Bi-variate analysis			Multi-variate analysis		
	Beta coefficient	p-value	cOR (95% CI)	Beta coefficient	p-value	aOR (95% CI)
Global intercept				0.225		1.252
intact cord (reference cord clamped)						
Intercept	0.133		1.143			
Intact cord	0.661	<0.0001	1.94 (1.56, 2.40)	0.609	<0.0001	1.84 (1.48, 2.29)
Complication during admission						
Intercept	1.034		2.814			
Yes	-0.701	<0.0001	0.50 (0.38, 0.64)	-0.676	<0.0001	0.51 (0.39, 0.66)
Mode of delivery (reference spontaneous birth)						
Intercept	1.304	0	3.683			
Instrumental delivery	-0.313	0.019	0.73 (0.56, 0.95)	-0.283	0.037	0.75 (0.58, 0.98)
Immediate drying after birth (reference no drying)						
Intercept	0.528	0.001	1.695			
Immediate drying after birth	0.45	0.008	1.57 (1.12, 2.19)	0.4	0.022	1.49 (1.06, 2.10)
Low Birth Weight (reference 2500 gram or more)						
Intercept	1.05	0	2.857			
Low Birth Weight	-0.459	0	0.63 (0.51, 0.78)	-0.383	0.003	0.68 (0.53, 0.88)
Preterm (reference 37 weeks or more)						
Constant	0.991	0	2.694			
Preterm1	-0.359	0.006	0.70 (0.54, 0.90)	0.041	0.8	1.04 (0.76, 1.43)

Confidential: For Review Only

27 June 2021

Response to first reviewer's comment

Comment 1# When reviewing this paper, one major issue arose. In table 1, the proportions are not calculated correctly. For example, labour was induced in 482/1892 infants who were stimulated after cord clamping, which is in my opinion 25.5% instead of the 79.5% which is noted in the Table. It looks like the authors have calculated the proportions based on the total number of infants born after induction of labour (482+124=606). However, since the group of infants stimulated with an intact cord is much smaller than the group of infants stimulated after cord clamping, this calculation of proportions is in my opinion not correct, since you would always get lower percentages in the smallest group. I would like to suggest adaption of this entire table.
In addition to this issue, statistics have to be recalculated, since there might be no significant differences with regard to these obstetrical and neonatal characteristics. Then, there would be no need to adjust for these variables.

Response- we have now re-calculated the proportion as column proportion in table 1.

Comment 2# One other comment about this study is about the performance of stimulation. How was tactile stimulation defined? Was this intervention standardized? Or did the execution of this intervention differ between caregivers/centers/clinical scenarios? Maybe the authors could add a little bit more detail about this intervention in their methods section.

Response- We have now defined it is study population in participation description in participant section.

Response to second reviewer's comment

Comment 3# In this observational study, the authors studied vaginally born infants who did not cry immediately after birth. They conclude that stimulation with the cord intact may be more effective in triggering spontaneous breathing and results in better Apgar scores than stimulation provided after cord clamping. However, I do not think that this conclusion is valid given the following.

It is possible (and arguably probable), that infants born in poor condition (floppy, pale) triggered greatest concern for the attending health worker who clamped the cord early to commence bag mask ventilation. Similarly, infants born following difficult labour (for example, prolonged bradycardia) and/or difficult birth (for example shoulder dystocia) may have led to greater anxiety for the attending health worker leading to early cord clamping. The predictor variable "cord clamping prior to stimulation" is therefore likely to be a reflection of the sicker infants in the cohort. Unsurprisingly, these infants had worse outcomes than infants who were stimulated with intact cord, where the health worker is likely to have been less concerned regarding the clinical status of the infant. This is supported by the finding that the proportion of infants with 1-minute Apgar score <4 (as reported in the text- see my comment below regarding data in Table 2) was greater in the cord clamped group.

It may theoretically be possible to try to answer the study question by statistically

adjusting for how unwell the infant is at birth (for example, in a regression model). However, this would require detailed assessment of the infant's condition immediately after birth, in addition to the antenatal/delivery factors. For example, tone, colour, heart rate. This data is difficult to measure, and for tone/colour, difficult to have standardised definitions that are not at risk of bias in an unblinded study. Fundamentally, I do not think the question of whether intact cord stimulation is associated with better outcomes (spontaneous breathing included) can be addressed with any validity outside a randomised trial.

While my own bias aligns with the authors' suggestion that intact cord stimulation is likely to be beneficial for most infants in primary apnea, the resulting delay in bag-mask-ventilation for the sicker infants is a critical factor to take into account. The balance of benefit for infants in primary apnea and harm for infants in secondary apnea who need bag-mask ventilation can only really be evaluated in a well conducted randomised trial. I am concerned that findings from observational studies that cannot realistically adjust for critical confounders may be used to inform resuscitation guidelines.

Response- We agree that neonates who that their cord intact had lesser complication during labour so might be less requiring bag-and-mask ventilation. However, we adjusted the complication during labour in table 3. We also agree that stimulation might have been effective more with primary apnea and for those with secondary apnea (complication during labour, dystocia) will require bag-and-mask ventilation. This hypothesis can only be answered through a randomized clinical trial.

Comment 4- I'm afraid I do not understand how the variables for inclusion in the regression model were selected. The authors state, "The multivariable analysis using logistic regression was done to assess the association of cord status during stimulation on breathing by adjusting for the variables which had a significant level difference of p<=0.01. The variables which were significantly different in cord status during stimulation were complications during admission, mode of delivery and immediate drying."

Firstly, how were variable selected for uni-variable comparison with the study outcome? For example, preterm and low birthweight are in the baseline characteristics table, but no data is provided on the univariable association with the study outcome. Were there any other variables collected and evaluated in the analysis, for example breech vaginal birth, shoulder dystocia, meconium stained liquor?
Secondly, the text suggests that variables were selected based on the association with the variable "cord status", rather than the study outcome "breathing after stimulation". Is this correct? The authors also state a univariable threshold of <=0.01 for inclusion in the multivariable model, but mode of delivery had p=0.21.

Response- We included only those variables or characteristics which were found to be significantly different (p-value<=0.01) two groups table 3. We have now adjusted preterm and low birth weight as it was significantly different in two groups. For breech birth, shoulder dystocia and meconium stained liquor, we have included them in complication during labour.

Comment 5. The following definitions require clarification. a) maternal complication during admission: any medical/obstetric complications during admission. It would be helpful to provide a list of complications.

Response- We have now added the definition of complication during admission in page 8, data collection and management.

Comment 6. The following definitions require clarification. b) Breathing after stimulation. How was this determined by the attending researcher? Was it simply not requiring bag-mask ventilation? If yes, it would be simpler to have bag-valve-mask ventilation as the study outcome.

Response- We have now added the definition of breathing after stimulation.

Comment 7. The percentages shown in Tables 1 and 3 are very difficult to follow. It would be more informative to show the values as in Table 2, where within each column, the total for a variable adds up to 100%.

Response- We have now revised table 1 and 3 as table 2.

Comment 8. Table 2, Apgar score in the 2 groups is inconsistent with the text. It appears here that the intact cord stimulation group had worse Apgar scores.

Response- Yes, we have now revised the typo.

Third reviewer's comment

Comment 9. Thank you for the opportunity to review this work. This is said to be an observational study assessing the impact of intact versus clamped umbilical cord on start of spontaneous breathing after stimulation of non-crying neonates in Nepal.
I commend the authors for the comprehensive work setting up these studies in Nepal. My main concern regarding this manuscript is that you do not report any specific variables related to the condition of the babies at birth, neither the timing of different actions/events from birth (i.e. how quickly did they start stimulation, when was cord clamping performed, initiation of breathing vs ventilation after birth). Due to the observational design and cord clamping on discretion of the midwives, one would suspect a great selection bias between the two groups (as long as you do not adjust for more specific variables as mentioned above). The midwives would likely tend to delay cord clamping and try stimulation among babies who seem less depressed, and thus more likely to respond to stimulation and start spontaneous breathing rather quickly. Therefore, I struggle with the conclusion, lines 43-57 page 13: "In conclusion, our study found offer non-crying neonates stimulated with the cord intact more like to breathe than those who are stimulated with cord clamped." I think the language could be improved several places throughout the manuscript.

Response- We have described the condition of the neonates at birth especially how quickly they start stimulation and cord clamping performed. We agree that midwives ted of delay cord clamping and try to stimulate babies who are less depressed, so respond to stimulation and spontaneously breathe. So, our conclusion is now "Our study found, among the neonates who are non-crying neonates, possibly with primary apnea, stimulated with cord intact more like to breathe than those who are stimulated with cord clamped."

Confidential: For Review Only

Comment 10-Abstract. Since this is an observational study and you don't adjust for the babies' condition at birth, I think you should reframe this conclusion as well: "This study provides evidence on the effect of the intact cord during stimulation." I think you should be cautious with words like "evidence" and "effect".

Response- We think that this study provides new evidence of effect of intact cord during stimulation, so would like to keep the conclusion as it is.

Comment 11-Methods. Study sites: Could you describe the resuscitation equipment/bay at the study sites? You state that the health care workers were trained to resuscitate with intact cord.

Response- We have now described the resuscitation equipment in the hospitals and health workers trained to resuscitate with intact cord in the study setting.

Comment 12-Participants: In the abstract you say ≥ 34 weeks. Is it >33 or ≥ 34?

Response- It is ≥ 34 weeks, we have made it consistent.

Comment 13- Variables: You did not include any variables that could indicate the state/condition of the baby at birth? For example, the final fetal heart rate and/or immediate newborn heart rate? Did you not include a variable to describe whether the baby was ventilated or not?

Response- We donot measure newborn heart rate after birth. We did include variable to describe baby was ventilated or not.

Comment 14- Data collection: Were all deliveries observed by a clinical researcher? 24/7? If not, is there a potential selection bias? How do you know if stimulation happened before or after cord clamping – this is not explained in detail? I also think the timelines are essential – when did cord clamping happen in the two groups (e.g. from time of birth) in relation to stimulation and start of breathing or ventilation. Do you have data on that? I think these data together with more information about the babies' status at birth are essential to better understand a potential impact of stimulation with intact cord.

Response- We have now provided the timing of cord clamping between two groups.

Comment 15-Results. It took me a while to understand table 1. For me it was intuitive (because of your design) to summarize the rows under each subheading for the two groups/columns (clamped vs intact). Maybe you should consider explaining a bit more how to read it, e.g. insert the denominator (n=...) for each row? What is reported in row 1? It is a bit confusing because you state in the methods that you are comparing the two groups (intact vs clamped cord), but in the result section you report differences among those being stimulated with intact cord for different obstetric and neonatal characteristics? "Among the neonates whose mother had complications during admission, intact cord during stimulation was lower than if mother had no complication (20.6% vs 26.9%, p-value=0.022)" etc.

Response- We have now revised the table 1.

Confidential: For Review Only

Comment 16- Table 2, I think there are some mistakes in the cut-off definitions of Apgars? And it is not consistent with the text. When (after birth) and how is mortality recorded? How is a stillborn defined?
It may be okey to state that there is a 94% higher odds of breathing if the cord was intact etc..
But I think it is more common (and precise/correct) to say that the odds of starting breathing versus not starting breathing increased 1,94 fold in the group with intact cord during stimulation.

Response- We have now defined stillborn. We have revised the sentence as "odds of starting breathing versus not starting breathing increased 1,94 fold in the group with intact cord during stimulation."

Comment 17- Discussion Lines 38-57 at page 11; I am not sure how "similar" (and/or relevant) the studies you report here are to your study. They seem to include premature babies who required ventilation (CPAP and stabilisation or PPV as part of resuscitation?). Gestational age is not reported in your study, but from table 1, it seems to be mostly term babies.

Response- Yes, our study does report gestational age now in table 3, so can be said similar.

Comment 18- Lines 3-13 page 12; "…effect on delayed versus cord clamping"? Please explain what you mean. What was defined as "resuscitation measures performed" in this reference 19? Again, how "similar" is this study to your present study?

Response- The study population to reference 19 was led by our team. The study population was similar to the one included in the study.

Comment 19- Lines 24-30 page 12; Reference 23 investigates the relationship between time to cord clamp and onset of breathing or initiation of PPV following stimulation/suction, AND 24-hour outcome. Time to start ventilation (among those who receive it) is critical. Reference 39 does not investigate if there is "an association between time to cord clamping and onset of breathing or initiation of PPV following stimulation/suction" as you state.

Response- We agree that Tanzania study, showed a contradictory result that timing of cord clamping is not of value, so was referenced here. We have now removed reference 25, 26 and 29.

Comment 20- Overall, I think the discussion section is difficult to follow and often unclear to me. Several places, I struggle to see/understand the relevance/relation to what your data potentially show/indicate. Hopefully you could explain this in more detail.

Response- We have re-edited the discussion section.

Confidential: For Review Only

BMJ Paediatrics Open

Impact of stimulation among non-crying neonates with intact cord versus clamped cord on birth outcomes-observation study

Journal:	*BMJ Paediatrics Open*
Manuscript ID	bmjpo-2021-001207.R1
Article Type:	Original research
Date Submitted by the Author:	31-Aug-2021
Complete List of Authors:	KC, Ashish; Uppsala University, Department of Women's and Children's Health<br>Budhathoki, Shyam; Imperial College London, Department of Primary Care and Public Health; Golden Community<br>Thapa, Jeevan; Patan Academy of Health Sciences, Department of Community Health Sciences<br>Niermeyer, Susan; University of Colorado Denver School of Medicine, Pediatrics<br>Gurung, Rejina; Uppsala University, Department of Women's and Children's Health; Golden Community<br>Singhal, Nalini; University of Calgary, Pedaitrics
Keywords:	Resuscitation, Epidemiology, Neonatology

SCHOLARONE™
Manuscripts

I, the Submitting Author has the right to grant and does grant on behalf of all authors of the Work (as defined in the below author licence), an exclusive licence and/or a non-exclusive licence for contributions from authors who are: i) UK Crown employees; ii) where BMJ has agreed a CC-BY licence shall apply, and/or iii) in accordance with the terms applicable for US Federal Government officers or employees acting as part of their official duties; on a worldwide, perpetual, irrevocable, royalty-free basis to BMJ Publishing Group Ltd ("BMJ") its licensees and where the relevant Journal is co-owned by BMJ to the co-owners of the Journal, to publish the Work in this journal and any other BMJ products and to exploit all rights, as set out in our licence.

The Submitting Author accepts and understands that any supply made under these terms is made by BMJ to the Submitting Author unless you are acting as an employee on behalf of your employer or a postgraduate student of an affiliated institution which is paying any applicable article publishing charge ("APC") for Open Access articles. Where the Submitting Author wishes to make the Work available on an Open Access basis (and intends to pay the relevant APC), the terms of reuse of such Open Access shall be governed by a Creative Commons licence – details of these licences and which Creative Commons licence will apply to this Work are set out in our licence referred to above.

Other than as permitted in any relevant BMJ Author's Self Archiving Policies, I confirm this Work has not been accepted for publication elsewhere, is not being considered for publication elsewhere and does not duplicate material already published. I confirm all authors consent to publication of this Work and authorise the granting of this licence.

Confidential: For Review Only

Impact of stimulation among non-crying neonates with intact cord versus clamped cord on birth outcomes- observation study

Ashish KC[1], Shyam Sundar Budhathoki[2,3], Jeevan Thapa[4], Susan Niermeyer[5], Rejina Gurung[1,3], Nalini Singhal[6] on behalf of Nepal Neonatal Network[ø]

[1]Department of Women's and Children's Health, Uppsala University, Uppsala, Sweden;

[2]Department of Primary Care and Public Health, School of Public Health, Imperial College London, London, United Kingdom;

[3]Golden Community, Lalitpur, Nepal;

[4] Department of Community Health Sciences, Patan Academy of Health Sciences, Lalitpur, Nepal;

[5]University of Colorado School of Medicine, Colorado School of Public Health, Aurora, CO, USA;

[6]Department of Pediatrics, University of Calgary, Calgary, Canada.

Correspondence- Ashish KC, Department of Women's and Children's Health, Uppsala University, SE-751 85, Uppsala, Sweden, www.kbh.uu.se/imch. Email address: ashish.k.c@kbh.uu.se; Phone number: +46 0729001074

Word count- 2533

Confidential: For Review Only

ABSTRACT

Background

Stimulation of non-crying neonates after birth can help transition to spontaneous breathing. In this study, we aim to assess the impact of intact versus clamped umbilical cord on spontaneous breathing after stimulation of non-crying neonates.

Methods

This is an observational study among non-crying neonates (n=3,073) born in hospitals of Nepal. Non-crying neonates born vaginally at gestational age ≥34-weeks were observed for their response to stimulation with the cord intact or clamped. Obstetric characteristics of the neonates were analysed. Association of spontaneous breathing with cord management was assessed using logistic regression.

Results

Among non-crying neonates, 2,563 received stimulation. Of these, a higher proportion of the neonates were breathing in the group with cord intact as compared to the group cord clamped (81.1% vs 68.9%, $p<0.0001$). The use of bag-and-mask ventilation was lower among those who were stimulated with the cord intact than those who were stimulated with cord clamped (18.0% vs 32.4%, $p<0.0001$). The proportion of neonates with Apgar-score ≤3 at 1 minute was lower with the cord intact than with cord clamped (7.6% vs 11.5%, $p=0.001$). In multi-variate analysis, neonates with intact cord had 84%

Confidential: For Review Only

increased odds of spontaneous breathing (aOR, 1.84; 1.48, 2.29) compared to those with cord clamped.

Conclusions

Stimulation of non-crying neonates with intact cord was associated with more spontaneous breathing than among infants who were stimulated with cord clamped. Intact cord stimulation may help establish spontaneous breathing in apnoeic neonates, but residual confounding variables may be contributing to the findings. This study provides evidence for further controlled research to evaluate the effect of initial steps of resuscitation with cord intact.

Key words: non-crying neonates, stimulation with cord intact, spontaneous breathing, resuscitation.

Confidential: For Review Only

What is already known on this topic?

- Stimulation is one of the key steps to neonatal resuscitation
- Breathing prior to umbilical cord clamping has been shown to result in smoother cardiovascular transition at birth in experimental studies

What this study adds?

- Intact cord stimulation to breathe with the cord intact may help deliver intervention more quickly than stimulation after cord clamping and may also avoid the reflex bradycardia that can occur when the cord is clamped before the lungs are aerated. Further research is needed to define if the results are confounded by factors such as provider experience or the baby's tone and colour influencing management decisions.

Confidential: For Review Only

INTRODUCTION

Globally, among the 140 million neonates who are born every year, 10-15 million do not cry or breathe at birth[1]. These neonates may require resuscitation to transition from the intrauterine to the extra-uterine environment[2]. Neonates who do not receive timely and adequate resuscitation may die or suffer brain injuries and long-term disability[3]. Every year, an estimated 1 million neonates die due to intrapartum-related complications or "birth asphyxia"[4], 2 million experience hypoxic-ischemic encephalopathy and 1.2 million go on to show developmental delay[3]. Most of this mortality and morbidity occur in low- and middle-income countries.

Transitioning to an extra-uterine environment depends on two major physiological events, the commencement of breathing and the transition of blood flow from the umbilical to the pulmonary circulation[2 5]. The trigger of breathing at birth results in clearance of the liquid in the trachea and airways, aeration of the lungs, reduction in pulmonary vascular resistance, and increase in pulmonary blood flow[6 7]. This increase in pulmonary blood flow supplies left ventricular preload previously dependent on flow across the patent foramen ovale from the right atrium and the umbilical vein[2]. Any intrapartum insult due to placental insufficiency (hypertension, infection or haemorrhage) can delay the spontaneous cardiopulmonary transition and may be compounded by umbilical cord clamping[8 9]. As a result, blood flows continuously through the ductus arteriosus, by-passing the pulmonary circulation into the systemic

Confidential: For Review Only

circulation as right-to-left shunting[6]. Resuscitation with cord intact may provide a smoother cardiopulmonary transition at birth.

The International Liaison Committee on Resuscitation provides guidance on neonatal resuscitation[10]. The ILCOR 2020 recommends that a neonate who is not crying or breathing with poor tone and heart rate less than 100 bpm should receive stimulation and clearing of airways (as needed) with intact cord[10]. There is very little robust evidence on the impact of stimulation in neonates receiving resuscitation with the cord intact. The World Health Organization (WHO) recommends if there is experience in providing ventilation without cutting the umbilical cord, ventilation can be initiated before cord-cutting.[11] WHO identifies resuscitation with the cord intact as a key research area to generate a stronger evidence base for care.[11].

Studies from low and middle-income countries have reported that neonates who are not crying or breathing at birth are immediately taken to a neonatal resuscitation area for stimulation, clearing of airways and positive-pressure ventilation[12 13]. Despite educational programs such as Helping Babies Breathe (HBB) from the American Academy of Pediatrics, which advocates stimulation to breathe before clamping the cord, health workers often clamp the cord before initiating resuscitation [14].

This study aims to evaluate the impact of stimulation among neonates with the cord intact versus those with cord clamped on breathing and birth outcome.

METHODS:

https://mc.manuscriptcentral.com/bmjpo

Confidential: For Review Only

Study Design: This was a nested observational study within two quality improvement studies SUSTAIN[15] and REFINE[16] done in nine hospitals of Nepal.

Study Sites: This study was conducted among nine hospitals in Nepal. The hospitals are tertiary care hospitals providing referral obstetric services through Comprehensive and Emergency Obstetric and Neonatal Care Services. These hospitals were chosen for quality improvement studies to implement a safer birth bundle. Health workers were trained on HBB 2nd edition to resuscitate neonates with intact cord when possible as part of the safer birth bundle. The cord management was at the discretion of the individual health worker.

Study Dates: The study was conducted between 1 June 2019 to 2 May 2020.

Participants: Women in labour with gestational age ≥34 weeks and confirmed fetal heart rate during admission were approached for consent. Women who were admitted for vaginal birth were approached for consent. For this study, neonates who were crying at birth were not eligible for analysis. Non-crying neonates who received additional tactile stimulation constituted the study population. Babies who cried immediately after birth and those with birth defects were excluded from the analysis. Neonates who did not cry and were not stimulated also were excluded from the study.

Sample size: All non-crying neonates who received stimulation were considered for analysis.

Variables: The variables considered were defined as cord status: intact and clamped; parity: no previous birth, 1 previous birth and 2 or more previous births; obstetrical

https://mc.manuscriptcentral.com/bmjpo

complication during admission: includes pre-eclampsia, eclampsia, premature rupture of membranes, pre-term labour, polyhydramnios, oligohydramnios, breech / transverse lie, decreased fetal movements, ante-partum haemorrhage, chorio-amnionitis, cord prolapse, meconium-stained amniotic fluid and maternal medical complications such as diabetes mellitus and pre-existing cardio-vascular and renal disease; induction of labour, augmentation labour; mode of delivery: instrumental (for forceps, vacuum application) or spontaneous; preterm birth (age of gestation less than 37 weeks); low birth weight (weight of baby less than 2500 gm); immediate thorough drying after birth and breathing after stimulation (spontaneous breathing requiring no positive-pressure ventilation). Stimulation was defined as additional rubbing of the back of non-crying neonates after drying. Stillbirth defined as no heart rate or breathing at any time after birth.

Data collection and Management: The data collection was done through the existing data collection systems of the two studies REFINE and SUSTAIN. Observational data was collected by independent clinical researchers using a tablet-based application (supplementary file 1). Observation was done on immediate newborn care, status of crying and when drying, clearing of the airway, stimulation, and cord clamping were performed. Researchers also extracted information from patient notes on obstetric complications, progress of labour, mode of delivery, sex and weight of neonate. m The data was then extracted into SPSS software for cleaning, coding and analysis.

Confidential: For Review Only

Data Analysis: The non-crying babies included in the study were categorised as those who were stimulated with intact cord and those who were stimulated after cord clamping. Spontaneous breathing after stimulation was observed. Obstetrical and neonatal characteristics were compared between the two groups.

Multivariable analysis using logistic regression was done to assess the association of cord status during stimulation on breathing by adjusting for the variables which had a significance level difference of $p \leq 0.01$. The variables which were significantly different by cord status during stimulation were complications during admission, mode of delivery and immediate drying.

Patients and public involvement- The main study aimed to improve the provision and experience of care of patients (mother and newborn) during childbirth. Information was provided to patients on their involvement in the main study. Results from this study will be disseminated to health care providers, paediatric associations and global neonatal guideline development organizations.

Ethical Approval: Ethical approval was granted by the Ethical Review Board of the Nepal Health Research Council for SUSTAIN (ref. 110/2019) and REFINE studies (ref. 87/2018). Written informed consent was obtained from the participants at the time of admission to the hospital before enrolling in the study.

RESULTS:

During the study period, 41,621 women delivered in the hospitals and 34,652 women consented to the study. Among the consented women 27,652 births were observed in

Confidential: For Review Only

the study. Among the total births observed 24,071 babies were excluded as they cried immediately after birth, and 508 births were further excluded due to birth defects; 3073 non-crying neonates were included in the analysis. Among the non-crying neonates, 2563 were stimulated. Of the neonates stimulated, 671 (26.2%) of them had their cord intact while 1892 (73.8%) had their cord clamped (figure 1).

We compared the obstetric and neonatal characteristics of neonates who had cord intact versus cord clamped before stimulation. Obstetric complications during admission were lower among neonates who had cord intact than those who had the cord clamped (8.0% vs 11.2%, p=0.022).

Neonates who had cord intact had lower proportion of breech or transverse lie during labour than those who had the cord clamped (1.3% vs 3.5%, p=0.003). Among neonates with cord intact a lower proportion had meconium- stained amniotic fluid during labour as compared to those with the cord clamped (22.2% vs 28.4%, p=0.002). Induction of labour was lower among neonates with cord intact than clamped (18.5% vs 25.5%, p<0.0001). The proportion of neonates born through instrumental delivery was lower among those with cord intact clamped (8.4% vs 12.3%, p=0.005) (Table 1).

The clinical outcome differed among neonates stimulated with the cord intact vs with those cord clamped. Among neonates stimulated with the cord intact, 81.1% breathed, while 68.9% breathed after stimulation with cord clamped (p-value <0.0001). The use of bag-and-mask ventilation was lower among those who were stimulated with the cord intact than those who were stimulated with cord clamped (18.0% vs 32.4%, p

Confidential: For Review Only

<0.0001). The proportion of neonates with Apgar score 3 or less at 1 minute was lower with the cord intact than clamped (7.6% vs. 11.5% p = 0.001). The proportion of neonates with Apgar score 6 or less at 5 minutes was lower with the cord intact than clamped (7.9% vs. 12. 7%, p < 0.0001). The proportion of mortality not significantly different between those with cord intact and clamped (0.7 vs 1.6%, p= 0.123) (Table 2).

We compared the obstetric and neonatal characteristics of non-crying neonates who breathed after stimulation. 58.3% of neonates with a maternal complication during admission breathed after stimulation, while 73.8% of neonates without maternal complication breathed after stimulation (p-value <0.0001). 66.3% Of those neonates born through instrumental delivery, 66.3% breathed after stimulation, while 72.9% of neonates who were born through spontaneous delivery breathed after stimulation (p-value=0.021). Among neonates who were not immediately dried after birth, 62.8% breathed after stimulation, while 72.7% of neonates who were immediately dried after birth breathed after additional stimulation (p-value=0.01). (Table 3)

In bivariate analysis, there were 1.94 higher odds of breathing if the cord was intact during stimulation compared to neonates with cord clamped (cOR, 1.94; 95% CI; 1.56, 2.40). If the neonate's mother had complications during admission for delivery, the odds of breathing after stimulation were 0.50 lower than neonate's whose mother had no complication during admission (cOR, 0.50; 95% CI; 0.38, 0.64). Neonates who were dried immediately after birth had 1.57 higher odds of breathing after stimulation than

Confidential: For Review Only

those who were not dried after birth (cOR, 1.57; 95% CI; 1.12, 2.19). In neonates with birth weight <2500 grams, the odds of breathing after stimulation was 0.63 lower than neonate's with low birth weight (cOR, 0.63; 95% CI; 0.51, 0.78). (Table 4)

In multi-variate analysis, to assess the association of characteristics with breathing after stimulation, there was 1.84 higher odds of breathing if the cord was intact during stimulation when compared to neonates with cord clamped (aOR, 1.85; 95% CI; 1.48, 2.30) after adjusting for obstetrical complications during admission, mode of delivery, immediate drying after birth, low birth weight and preterm birth. Neonates who were dried immediately after birth had 1.49 higher odds of breathing after stimulation than those who were not dried after birth (aOR, 1.47; 95% CI; 1.04, 2.07). (Table 4)

DISCUSSION

In this observational study, one-fourth of the non-crying neonates had stimulation performed with an intact cord. There were higher odds of spontaneous breathing if the cord was kept intact during stimulation than neonates who had their cord clamped during stimulation. The higher obstetrical complications during admission and Apgar score less than 4 at 1 minute among early cord clamped non-crying neonates indicates that these are more depressed neonates than neonates with cord intact. In line with this, a higher proportion of infants with early cord clamping received bag-and-mask ventilation. Even though there was no significant difference in mortality between the two groups, the mortality trended lower among the neonates who were stimulated with the cord intact.

Confidential: For Review Only

An experimental animal study by Bhatt et al. demonstrated a more stable cardiovascular adaptation if cord clamping was performed after initiation of ventilation[7]. The results from this and other studies suggested that resuscitation of newborns should be performed with an uncut cord, facilitating the postnatal transition[17-19]. However, in a single-center observation study in Tanzania, there was no significant association between time to cord clamping and onset of breathing or initiation of positive-pressure ventilation following stimulation/suction[20].

Similar to our results, a single-center randomized controlled trial (RCT) in California, US to evaluate the effect of delayed cord clamping in premature infants (<32 weeks) who required ventilation demonstrated that provision of gentle tactile stimulation with the cord intact may hasten the establishment of spontaneous respirations and provide a similar placental transfusion compared to positive-pressure ventilation with delayed cord clamping.[21] Another multi-centric trial in the UK among premature infants (≤ 32 weeks) suggests that cord clamping after at least 2 min and providing neonatal care with the cord intact may improve outcome at discharge as compared to early clamping.[22]

A single-center RCT in Nepal evaluated the effect on delayed versus early cord clamping in babies requiring resuscitation[23]. In the trial, randomization was performed while the baby was still in utero, and resuscitation measures (stimulation and positive-pressure ventilation) performed according to the HBB algorithm with an unclamped cord in the mother's bed were associated with early spontaneous breathing.

https://mc.manuscriptcentral.com/bmjpo

Our multi-centric study conducted between 2017-2018 showed that 9.8% of neonates do not cry at birth[12]. The study found that non-crying but breathing babies might not have the full respiratory capacity and require resuscitation.[24] In this study, we hypothesize that the non-crying neonates who breathe spontaneously after stimulation might have been in primary apnea. There is increased oxygenation with intact cord due to several interacting mechanisms; persisting oxygenation by the placenta, avoidance of bradycardia often associated with clamping the cord before the onset of respirations, and earlier initiation of breathing resulting in increased pulmonary blood flow.

A study in Tanzania showed that although the health care provider commonly practiced clamping the umbilical cord immediately after delivery, they were aware that delayed cord clamping has a potential benefit of oxygenation to the newborn in the event of the need for resuscitation.[25] Our study also shows that despite the education of health care providers on keeping the cord intact during resuscitation, two-thirds of the non-crying babies received resuscitation after cord clamping. There is a need to implement a delay in clamping as a quality improvement intervention to improve initial steps of resuscitation -especially stimulation and if necessary bag-and-mask ventilation. Three recent systematic reviews and meta-analysis showed that there is a lack of evidence to recommend cord management among term and preterm infants who receive positive-pressure ventilation at birth.[26 27]

Methodological consideration

Confidential: For Review Only

This study has several strengths including that observations were done by trained researchers in multiple hospitals in Nepal in a consistent way and with large sample size. Using observational methods limited recall bias. However, there are several limitations. First, this observation was only done for vaginal births and not for caesarean births. Second, cord management was at the discretion of the health workers, so there may have been a bias toward the previous practice of immediate cord clamping. The appearance of the baby at birth (degree of cyanosis, hypotonia) may have influenced decision-making on when to cut the cord

In conclusion, our study found that non-crying neonates stimulated with the cord intact were more like to breathe than those who were stimulated with the cord clamped. Stimulation with cord intact may help establish spontaneous breathing in apnoeic neonates, but residual confounding variables are likely contributing to the findings. Our study provides additional evidence consistent with the ILCOR recommendation on keeping the cord intact during the initial steps of resuscitation. This study highlights the importance of further controlled research to evaluate the effect of initial steps of resuscitation with cord intact.

ᴓNepal Neonatal Network-Omkar Basnet, Srijana Sharma, Pratikshya Bhattarai, Ankit Acharya, Prof. Anjani Kumar Jha, Dr. Yasho Vardhan Pradhan, Dr. Sushil Nath Pyakurel, Prof. Kiran Bajracharya, Dr. Sheela Verma, Dr. Amit Bhandari, Dr. Anjana KC Thapa, Dr. Krishna Prasad Bista, Jhalak Sharma Paudel, Sangita Khatri, and Leela Paudel.

Confidential: For Review Only

Author's contribution-AKC and NS conceptualized the study. AKC, JT and SSB conducted the analysis. NS, RG provided feedback to the analysis. AKC, SSB, RG and JT wrote the first draft. NS and SN made the second and subsequent revisions to the draft. All authors reviewed and provided feedback on the revised version and agreed to the final version.

Conflict of Interest-We declare no conflict of interest

Consent for Publication-None

Availability of supporting data

The dataset generated and analysed is not publicly available as it is part of larger quality improvement projects but can be made available on reasonable request with a data-sharing agreement.

Competing interests-The authors declare that they have no competing interests.

Funding-No specific funding was available for this study.

Acknowledgement-We would like to thank all the data coordinators and surveillance officers, hospital manager, nursing in-charge, all the nurses and support staff working in the sick newborn care units. We thank all the mothers and caregivers of the sick newborns who consented to the study.

Confidential: For Review Only

References

1. Lee AC, Cousens S, Wall SN, et al. Neonatal resuscitation and immediate newborn assessment and stimulation for the prevention of neonatal deaths: a systematic review, meta-analysis and Delphi estimation of mortality effect. *BMC Public Health* 2011;11 Suppl 3:S12. doi: 10.1186/1471-2458-11-S3-S12 [published Online First: 2011/04/29]
2. Hooper SB, Polglase GR, te Pas AB. A physiological approach to the timing of umbilical cord clamping at birth. *Arch Dis Child Fetal Neonatal Ed* 2015;100(4):F355-60. doi: 10.1136/archdischild-2013-305703 [published Online First: 2014/12/30]
3. Lee AC, Kozuki N, Blencowe H, et al. Intrapartum-related neonatal encephalopathy incidence and impairment at regional and global levels for 2010 with trends from 1990. *Pediatr Res* 2013;74 Suppl 1:50-72. doi: 10.1038/pr.2013.206 [published Online First: 2013/12/25]
4. Liu L, Oza S, Hogan D, et al. Global, regional, and national causes of under-5 mortality in 2000-15: an updated systematic analysis with implications for the Sustainable Development Goals. *Lancet* 2016;388(10063):3027-35. doi: 10.1016/S0140-6736(16)31593-8 [published Online First: 2016/11/15]
5. Gao Y, Raj JU. Regulation of the pulmonary circulation in the fetus and newborn. *Physiol Rev* 2010;90(4):1291-335. doi: 10.1152/physrev.00032.2009 [published Online First: 2010/10/21]
6. Crossley KJ, Allison BJ, Polglase GR, et al. Dynamic changes in the direction of blood flow through the ductus arteriosus at birth. *J Physiol* 2009;587(Pt 19):4695-704. doi: 10.1113/jphysiol.2009.174870 [published Online First: 2009/08/14]
7. Bhatt S, Alison BJ, Wallace EM, et al. Delaying cord clamping until ventilation onset improves cardiovascular function at birth in preterm lambs. *J Physiol* 2013;591(8):2113-26. doi: 10.1113/jphysiol.2012.250084 [published Online First: 2013/02/13]
8. Polglase GR, Dawson JA, Kluckow M, et al. Ventilation onset prior to umbilical cord clamping (physiological-based cord clamping) improves systemic and cerebral oxygenation in preterm lambs. *PLoS One* 2015;10(2):e0117504. doi: 10.1371/journal.pone.0117504 [published Online First: 2015/02/18]
9. Katheria AC, Brown MK, Rich W, et al. Providing a Placental Transfusion in Newborns Who Need Resuscitation. *Front Pediatr* 2017;5:1. doi: 10.3389/fped.2017.00001 [published Online First: 2017/02/10]
10. Aziz K, Lee CHC, Escobedo MB, et al. Part 5: Neonatal Resuscitation 2020 American Heart Association Guidelines for Cardiopulmonary Resuscitation and Emergency Cardiovascular Care. *Pediatrics* 2021;147(Suppl 1) doi: 10.1542/peds.2020-038505E [published Online First: 2020/10/23]
11. World Health Organization. Guidelines on basic newborn resuscitation. In: WHO, ed. Geneva: WHO, 2012.
12. Kc A, Lawn JE, Zhou H, et al. Not Crying After Birth as a Predictor of Not Breathing. *Pediatrics* 2020;145(6) doi: 10.1542/peds.2019-2719 [published Online First: 2020/05/14]
13. Kc A, Ewald U, Basnet O, et al. Effect of a scaled-up neonatal resuscitation quality improvement package on intrapartum-related mortality in Nepal: A stepped-wedge cluster randomized controlled trial. *PLoS Med* 2019;16(9):e1002900. doi: 10.1371/journal.pmed.1002900 [published Online First: 2019/09/10]
14. Kamath-Rayne BD, Thukral A, Visick MK, et al. Helping Babies Breathe, Second Edition: A Model for Strengthening Educational Programs to Increase Global Newborn Survival. *Glob Health Sci Pract* 2018;6(3):538-51. doi: 10.9745/GHSP-D-18-00147 [published Online First: 2018/10/06]
15. Gurung R, Jha AK, Pyakurel S, et al. Scaling Up Safer Birth Bundle Through Quality Improvement in Nepal (SUSTAIN)-a stepped wedge cluster randomized controlled trial in public hospitals. *Implement Sci* 2019;14(1):65. doi: 10.1186/s13012-019-0917-z [published Online First: 2019/06/21]

Confidential: For Review Only

16. Gurung R, Gurung A, Basnet O, et al. REFINE (Rapid Feedback for quality Improvement in Neonatal rEsuscitation): an observational study of neonatal resuscitation training and practice in a tertiary hospital in Nepal. *BMC Pregnancy Childbirth* 2020;20(1):756. doi: 10.1186/s12884-020-03456-z [published Online First: 2020/12/05]
17. Niermeyer S, Velaphi S. Promoting physiologic transition at birth: re-examining resuscitation and the timing of cord clamping. *Semin Fetal Neonatal Med* 2013;18(6):385-92. doi: 10.1016/j.siny.2013.08.008 [published Online First: 2013/09/24]
18. Mercer JS, Erickson-Owens DA. Is it time to rethink cord management when resuscitation is needed? *J Midwifery Womens Health* 2014;59(6):635-44. doi: 10.1111/jmwh.12206 [published Online First: 2014/10/10]
19. Bjorland PA, Ersdal HL, Eilevstjonn J, et al. Changes in heart rate from 5 s to 5 min after birth in vaginally delivered term newborns with delayed cord clamping. *Arch Dis Child Fetal Neonatal Ed* 2020 doi: 10.1136/archdischild-2020-320179 [published Online First: 2020/11/12]
20. Ersdal HL, Linde J, Auestad B, et al. Timing of cord clamping in relation to start of breathing or ventilation among depressed neonates-an observational study. *BJOG* 2016;123(8):1370-7. doi: 10.1111/1471-0528.13778 [published Online First: 2015/12/25]
21. Katheria A, Poeltler D, Durham J, et al. Neonatal Resuscitation with an Intact Cord: A Randomized Clinical Trial. *J Pediatr* 2016;178:75-80 e3. doi: 10.1016/j.jpeds.2016.07.053 [published Online First: 2016/10/30]
22. Duley L, Dorling J, Pushpa-Rajah A, et al. Randomised trial of cord clamping and initial stabilisation at very preterm birth. *Arch Dis Child Fetal Neonatal Ed* 2018;103(1):F6-F14. doi: 10.1136/archdischild-2016-312567 [published Online First: 2017/09/20]
23. Andersson O, Rana N, Ewald U, et al. Intact cord resuscitation versus early cord clamping in the treatment of depressed newborn infants during the first 10 minutes of birth (Nepcord III) - a randomized clinical trial. *Matern Health Neonatol Perinatol* 2019;5:15. doi: 10.1186/s40748-019-0110-z [published Online First: 2019/09/06]
24. David G Tingay, Olivia Farrell, Jessica Thomson, et al. Imaging the Respiratory Transition at Birth: Unravelling the Complexities of the First Breaths of Life. *American Journal of Respiratory and Critical Care Medicine* 2021 February (accepted)
25. Mwakawanga DL, Mselle LT. Early or delayed umbilical cord clamping? Experiences and perceptions of nurse-midwives and obstetricians at a regional referral hospital in Tanzania. *PLoS One* 2020;15(6):e0234854. doi: 10.1371/journal.pone.0234854 [published Online First: 2020/06/23]
26. Jeevan A, Ananthan A, Bhuwan M, et al. Umbilical cord milking versus delayed cord clamping in term and late-preterm infants: a systematic review and meta-analysis. *J Matern Fetal Neonatal Med* 2021:1-11. doi: 10.1080/14767058.2021.1884676 [published Online First: 2021/02/12]
27. Seidler AL, Gyte GML, Rabe H, et al. Umbilical Cord Management for Newborns <34 Weeks' Gestation: A Meta-analysis. *Pediatrics* 2021;147(3) doi: 10.1542/peds.2020-0576 [published Online First: 2021/02/27]

Confidential: For Review Only

Tables and figures

Table 1. Obstetric and neonatal characteristics among non-crying neonates with intact cord versus cord clamped during stimulation

	Intact cord during stimulation n-671	Cord clamped before stimulation n-1892	p-value
Median time to cord clamping (IQR) seconds	58.0 (34, 71)	25 (18, 31)	
Parity			
Nullipara, 1430	376 (56.5%)	1054 (56.0%)	0.892
Primipara, 714	180 (27.0%)	534 (28.4%)	0.515
Multipara, 403	110 (16.5%)	293 (15.6%)	0.579
Complication during admission			
No, 2292	617(92.0%)	1675(88.8%)	
Yes, 266	54 (8.0%)	212 (11.2%)	0.022
Antepartum haemorrhage, 5	5 (0.3%)	0 (0.0%)	0.335
Decreased foetal movements, 21	14 (0.7%)	7 (1.0%)	0.459
Pre-eclampsia and eclampsia, 49	34 (1.8%)	15 (2.24%)	0.291
Breech or transverse lie, 75	66 (3.5%)	9 (1.3%)	0.003
Prolapsed cord, 5	4 (0.2%)	1 (0.1%)	0.998
Chorio-amnionitis, 18	12 (0.6%)	6 (0.9%)	0.59
Premature rupture of membrane, 43	38 (2.0%)	5 (0.7%)	0.034
Preterm labour, 87	68 (3.6%)	19 (2.8%)	0.387
Oligohydramnios, 28	18 (1.0%)	10 (1.5%)	0.279
Medical complication*, 79	50 (2.6%)	29 (4.3%)	0.037
Meconium Stained fluid			
No, 1876	1354 (71.6%)	522 (77.8%)	
Yes, 687	538 (28.4%)	149 (22.2%)	0.002
Induction of labour			
No, 1957	547 (81.5%)	1410(74.5%)	
Yes, 606	124(18.5%)	482(25.5%)	<0.0001
Augmentation of labour			
No, 1600	445(66.3%)	1155(61.0%)	
Yes, 963	226(33.7%)	737(39.0%)	0.016
Mode of delivery			
Spontaneous, 2260	611(91.6%)	1649(87.7%)	
instrumental, 288	56(8.4%)	232(12.3%)	0.005
Gestational age in weeks (SD)	38.4±2.2	38.5±2.3	
Preterm (<37 weeks)			
No, 2272	594(88.5%)	1678(88.7%)	
Yes, 291	77(11.5%)	214(11.3%)	0.944
Birth weight in weeks (SD)	2784.3±543.7	2807.1±556.6	
Low birth weight (<2500 gram)			

https://mc.manuscriptcentral.com/bmjpo

No, 2083	537(82.3%)	1546(82.3%)	
Yes, 463	131(19.6%)	332(17.7%)	0.268
Immediate drying			
No, 159	35(5.2%)	124(6.6%)	
Yes, 2404	636(94.8%)	1892(93.4%)	0.227

Confidential: For Review Only

Table 2. Clinical outcome of non-crying neonates who were stimulated with cord intact vs cord clamped

	Intact cord during stimulation n-671 (26.2%)	Cord clamped before stimulation n-1892 (73.8%)	p-value
Spontaneous breathing			
Yes, 1847	544 (81.1%)	1303 (68.9%)	
No, 716	127 (18.9%)	589 (31.1%)	<0.0001
Bag-and-mask ventilation			
No, 1829	550 (82.0%)	1279 (67.6%)	
Yes, 734	121 (18.0%)	613 (32.4%)	<0.0001
Apgar score at 1 minute			
4 or more, 2214	598 (92.4%)	1616 (88.3%)	
3 or less, 264	49 (7.6%)	215 (11.5%)	0.001
Apgar score at 5 minute			
7 or more, 2196	596 (92.1%)	1597 (87.3%)	
6 or less, 284	51 (7.9%)	233 (12.7%)	<0.0001
Birth outcome			
Live born, 2513	654 (97.5%)	1859 (98.3%)	
Stillborn, 50	17 (2.5%)	33 (1.7%)	0.198
Mortality			
No, 2528	666 (99.3%)	1862 (98.4%)	
Yes, 35	5 (0.7%)	30 (1.6%)	0.123

Confidential: For Review Only

Table 3. The obstetric and neonatal characteristics of non-crying neonates who responded to stimulation

	Breathing after stimulation (n=1847	Not breathing after stimulation (n=716)	p-value
Complication during admission			
No, 2292	1691(91.6%)	601(84.4%)	
Yes, 266	155(8.4%)	111(15.6%)	<0.0001
Induction of labour			
No, 1957	1406(76.1%)	551(77.0%)	
Yes, 606	441(23.9%)	165(23.0%)	0.679
Augmentation of labour			
No, 1600	1159(62.8%)	441(61.6%)	
Yes, 963	688(37.2%)	275(38.4%)	0.586
Mode of delivery			
Spontaneous, 2260	1648(89.6%)	612(86.3%)	
Instrumental, 288	191(10.4%)	97(13.7%)	0.021
Intact cord during stimulation			
No, 1892	1303(70.5%)	589(82.3%)	
Yes, 671	544(29.5%)	127(17.7%)	<0.0001
Drying after birth			
No, 159	100(5.4%)	59(8.2%)	
Yes, 2404	1747(94.6%)	657(91.8%)	0.01
Gestational age in weeks (SD)	38.53±2.0	38.2±2.7	
Preterm birth (<37 weeks)			
No, 2272	1657 (89.7%)	615 (85.9%)	
Yes, 291	190 (10.3%)	101 (14.1%)	0.008
Birth weights in gram (SD)	2836.9±523.9	2707.6±614.0	
Low Birth Weight (<2500 gram)			
No, 2083	1543 (83.8%)	540 (76.6%)	
Yes, 463	298 (16.2%)	165 (23.4%)	<0.0001

For confidential: For Review Only

Table 4. Bi-variate and multi-variate analysis of association between obstetrics and neonatal characteristics with breathing after stimulation

	Bi-variate analysis			Multi-variate analysis		
	Beta coefficient	p-value	cOR (95% CI)	Beta coefficient	p-value	aOR (95% CI)
Global intercept				0.225		1.252
intact cord (reference cord clamped)						
Intercept	0.133		1.143			
Intact cord	0.661	<0.0001	1.94 (1.56, 2.40)	0.613	<0.0001	1.85 (1.48, 2.30)
Complication during admission						
Intercept	1.034		2.814			
Yes	-0.701	<0.0001	0.50 (0.38, 0.64)	-0.572	<0.0001	0.57 (0.43, 0.75)
Mode of delivery (reference spontaneous birth)						
Intercept	1.304	0	3.683			
Instrumental delivery	-0.313	0.019	0.73 (0.56, 0.95)	-0.324	0.018	0.72 (0.55, 0.95)
Immediate drying after birth (reference no drying)						
Intercept	0.528	0.001	1.695			
Immediate drying after birth	0.45	0.008	1.57 (1.12, 2.19)	0.385	0.028	1.47 (1.04, 2.07)
Low Birth Weight (reference 2500 gram or more)						
Intercept	1.05	0	2.857			
Low Birth Weight	-0.459	<0.0001	0.63 (0.51, 0.78)	-0.383	0.003	0.68 (0.53, 0.88)
Preterm (reference 37 weeks or more)						
Constant	0.991	0	2.694			
Preterm1	-0.359	0.006	0.70 (0.54, 0.90)	0.041	0.80	1.04 (0.76, 1.43)

cOR-crude Odds Ratio; CI- confidence interval, aOR- adjusted Odds Ratio

Confidential: For Review Only

Figure 1. Study flow figure

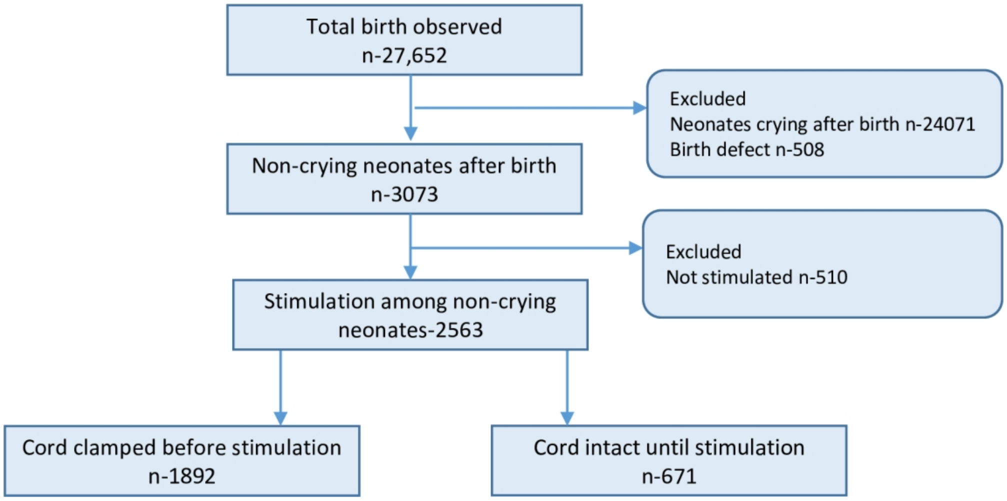

150x86mm (300 x 300 DPI)

ID NUMBER: ________________________

Form 1

SUSTAIN Registry Data

**Data Collector details**	Name	
	Code	

**Data ID**	**Information**		**Write or circle where applicable**	**Remarks**
**PART A: BACKGROUND INFORMATION**				
101.	Mother's first name			
102.	Mothers' last name			
103.	Mother's inpatient number			
104.	Age of mother (completed years)			
105.	Ethnicity code		Dalit………………………………………….1<br>Janajati………………………………………2<br>Madhesi……………………………………..3<br>Muslim………………………………………4<br>Brahmin/Chhetri……………………………..5<br>Others…………………………………………6	
106.	Address	Province		
		District		
		Municipality		
		Ward		
107.	Mobile			
108a	Place of referall for Delivery		Primary health centre…………………..1<br>District Hospital……………………….2<br>Health post…………………………….3<br>Other …………………………………4<br>NR……………………………………..99<br>Self……………………………………..5	
108.	Parity		Primipara (0 previous births)…………………….1	
			Multipara (2-5 births)……………………………2	
			Grand multipara (>5 births)…………………….3	

	Date (AD) dd/mm/yyyy	Signature
Form completed:		
Data entered into database:		

Confidential: For Review Only

PART B: PRE-DELIVERY DETAILS					
**Part B1: Complications during pregnancy (based on ANC records)**					
109.	**Complications recorded**	***Yes***	***No***	***NR***	
109a.	*Vaginal bleeding/APH*	1	0	99	
109b.	*Decreased foetal movements*	1	0	99	
109c.	*Eclampsia (including convulsions, coma, stroke or unconsciousness)*	1	0	99	
109d.	*Pre-eclampsia (BP >140/9mmHg and protein urine)*	1	0	99	
109e.	*Diabeties*	1	0	99	
109f.	*TORCHES*	1	0	99	
109g	*TORCHES (specify)……………………………………*				
109h.	*Others (specify) ……………………………………*				
110.	Place making referral				
111.	Anaemia	Yes………………..……………….1<br>No…………………………………..2<br>Not recorded…………..…….99			**If No or NR, go to 114**
112.	If Yes, type of anaemia	Mild (<11 gm/dL)…………….1<br>Moderate (7-10.9 gm/dL)…..2<br>Severe (<7 gm/dL)…………3			
113.	Cause of anaemia (multiple choice)	Iron deficiency………………1<br>Folic acid deficiency………..2<br>Vitamin B12 deficiency…….3<br>Sickle cell……………………4<br>Thalassemia…………………5<br><br>Others (specify)……………...			
Height	Height of the mother in cm	…………………….CM			
Weight	Weight of the mother from 1st ANC checkup	……………………….KG's			
**Part B2: Condition at the time of admission**					
114.	**Provisional diagnosis of any complication recorded at the time of admission**	***Yes***	***No***	***NR***	
114a.	*Antepartum haemorrhage (APH)*	1	0	99	
114b.	*Decreased foetal movements*				
114c.	*Prolonged labour (>12 hours active phase)*	1	0	99	
114d.	*Pre-eclampsia (BP >140/9mmHg and protein urine)*	1	0	99	
114e.	*Eclampsia (including convulsions, coma, stroke or unconsciousness)*	1	0	99	
114f.	*Breech or transverse lie*	1	0	99	
114g.	*Prolapsed cord*	1	0	99	
114h.	*Chorioamnionitis*	1	0	99	
114i.	*Premature rupture of membrane (PROM)*	1	0	99	
114j.	*Pre-term labour and/or preterm premature rupture of membranes (PPROM)*	1	0	99	

Confidential: For Review Only

114k.	*Foetal congenital anomaly*		1	0	99	
114l.	*Others (specify):*					
115.	Gestational age at admission by USG		[ ][ ] + [ ]<br>Not known………………………........0<br>Not recorded………………............99			
115A	Gestational age at admission by LMP		[ ][ ] + [ ]<br>Not known………………………........0<br>Not recorded………………............99			
Q115B	Anemia diagnosed during admission		Yes……………..………….…1<br>No………………………………....2<br>Not recorded………….…….99			
Q115C	If Yes, type of anaemia		Mild (<11 gm/dL)………….…1<br>Moderate (7-10.9 gm/dL)….2<br>Severe (<7 gm/dL)……….…3			
Q115D	Cause of anaemia (multiple choice)		Iron deficiency………….…1<br>Folic acid deficiency……..2<br>Vitamin B12 deficiency……3<br>Sickle cell………………….4<br>Thalassemia……………….5<br><br>Others (specify)……………6<br>recorded ………………..99			
116.	Mother's blood group		RH positive………………….…1<br>RH negative……………………2<br>Not recorded…..…....................99			
117.	ABO blood type		A………………………………1<br>B………………………………2<br>AB……………………………3<br>O……………………………4			
118.	FHR at admission		Yes, normal (100-160 bpm)……..1<br>Yes, abnormal (<110 or >160 bpm)..2<br>Absent………………...............3<br>Not recorded….........................99			**If No or NR, go to 120**
119.	If FHR recorded	Date (AD) dd/mm/yyyy	[ ][ ][ ][ ][ ][ ][ ][ ]			
		Time (hh:mm) 24-hr	[ ][ ][ ][ ]			
119B	Total Number of PV examinations					
120.	Stage of labour		Not in labour……………….….1<br>Latent stage of labour……......2<br>First stage of active labour…........3<br>Second stage of labour……..........4<br>Third stage of labour………..........5			
119A	Duration of active stage of labor		………………. hours			

PART C: DELIVERY DETAILS

Confidential: For Review Only

121.	Place of delivery	Home………………………..…......1<br>On the way …………………………......2<br>Health facility………….....……...…3	**If Home or On the way, go to 129**
Q121a	If Health facility	Inside delivery room……………..1<br>Outside delivery room…………2<br>NR………………………………………..99	
122.	Partograph use	Yes, completely filled……….…....1<br>Yes, partially filled………………...2<br>Not filled…………………………….…0	
123.	FHR monitoring recorded during delivery	Yes, as per protocol…………....…1<br>Yes, sporadically (> once)……….2<br>Yes, only once…………...……….…3<br>Not recorded…………………........99	
124.	Abnormal FHR detected during labour	Yes, bradycardia (< 110)…………..1<br>Yes, tachycardia (> 160)………….2<br>Yes, repetitive (during > 50% of contractions) or prolonged (> 3 min) decelerations……………….3<br>No……………………………..…………0<br>Not recorded…………………...99	
125.	Induction of labour	Induction with prostaglandins…....1<br>Induction with amniotomy……….2<br>Induction with oxytocin…………..3<br>No……………………………..…………0<br>Not recorded…………………...99	
126.	Augmentation of labour	Augmentation with oxytocin…....1<br>Augmentation with amniotomy....2<br>No……………………………..…………0<br>Not recorded…………………...99	

127.	Received prophylactic antibiotics	Yes…………………………..…………….....…………1<br>No…………………………………......................... 0	
127A	Corticosteroids given to mother<br>(Dexamethasone/Betamethasone)	Yes…………………………..…………….....…………1<br>No…………………………………......................... 0	
128.	Mode of delivery	Spontaneous vaginal………………......……….1<br>**Go to 131**<br>Instrumental……………......……................... 2<br>**Go to 130**<br>Manoeuvre delivery……………………………......3<br>**Go to 131**<br>Emergency CS………………………………….....4<br>Elective CS…………..………...................………5<br>**Go to 129**	

Confidential: For Review Only

129.	Reason for CS (multiple response)	Foetal distress (FD)……………………..………..1<br>Cephalopelvic disproportion (CPD)……………....2<br>Abnormal lie/malpresentation/ malposition...3<br>APH or intrapartum haemorrhage ………….….4<br>PIH (Pre-eclampsia/eclampsia)…………….…....5<br>Non-progress of labour (NPOL) or prolonged labour………………………………………..…..…...6<br>Multiple pregnancy………......................…......7<br>Cord around the neck……………………………….8<br>Cord prolapse…………………………..……...….9<br>Oligohydramnios……………………..………...…10<br>Previous CS……..…………………………...........….....11<br>Maternal request………………………………..…......12<br><br>Other (specify)………………………….……..…….…..	
130.	Reason for instrumental delivery	Prolonged labour…………..……………………....1<br>Foetal distress……..………………..……………...2<br>Maternal distress…………………..………………...3<br><br>Other (specify)……..…………..……..…………..….	
131.	Multiple delivery	Yes…………………………..…………….…………..1<br>No……………………………….................….... 0	
If multiple deliveries, use additional form for each baby!			
132.	Delivery conducted by	Nursing staff………………………………..……1<br>Doctor………………………..…………………..…. 2<br>Other health personnel…....…………………..3<br>Not recorded…………………………………..…99	
133.	Complication to mother before or at the time of delivery	Yes…………………………..…………..………..1<br>No……..……………………….…………..……...0<br>Not recorded…………………………………..…99	**If No or NR, go to 135**
134.	If Yes (multiple choice)	Manoeuvre delivery……………………..…………1<br>Prolonged labour………………………………….2<br>Antepartum haemorrhage……………………..3<br>Postpartum haemorrhage……………………..4<br>Pregnancy induced hypertension.. ………....5<br>Oligohydramnios……………………………….6<br><br>Others (specify)…………………………..………	

135.	Amniotic fluid	Clear……………………………………..…….1<br>Thin meconium stained………..…...2<br>Thick meconium stained………........3<br>Not recorded…………………..……...99	
136.	Complication to baby at the time of delivery	Yes………………………………………….1<br>No………………………..………………..0<br>Not recorded………………..…………...99	**If No or NR, go to 138**
137.	If Yes (multiple choice)	Birth asphyxia……………………………...1<br>Grunting……………………..…………..…..2<br>Foetal distress………....…..…..……3<br>Preterm………..…..…………………..…..4<br>Meconium stained…………………..…....5<br>Tachypnoea…………....…..………..…. 6	

Confidential: For Review Only

Confidential: For Review Only

		Breech delivery……………..…..7<br>Low birth weight……………..….8<br>Sepsis……………..……………..9<br>Cyanosis……………..……………10<br>Poor cry……………..……………11<br>Respiratory distress syndrome……12<br>Big baby……………………………13<br>Gasping ……………………..…..…14<br>Others (specify)……………..………			
138.	Date of delivery (AD) dd/mm/yyyy	☐☐☐☐☐☐☐☐			
139.	Time of delivery (hh:mm) 24-hr	☐☐☐☐			
140.	Sex of baby	Girl…………………………........ .....1<br>Boy………………...............................0			
141.	Birth weight (grams)	☐☐☐☐			
142.	Gestational age by LMP (weeks)	☐☐ + ☐<br>Not known…………………......... 0<br>Not recorded………………............99			
143.	Delivery outcome	Livebirth……………..…………......1<br>Stillbirth………..……………………2			**If Livebirth, go to 145**
144.	If stillbirth, type of stillbirth	Fresh……………………………..…..1<br>Macerated……………………........ 2			**End extraction!!!**
145.	APGAR at 1 minute	☐☐			
146.	APGAR at 5 minutes	☐☐			
147.	**Method of Resuscitation**	***Yes***	***No***	***NR***	
147a.	Clearing of airway (suctioning)	1	0	99	
147b.	Stimulation	1	0	99	
147c.	Bag-mask ventilation	1	0	99	
	Other interventions				
147d.	Oxygen	1	0	99	
147e.	Medication	1	0	99	
147f.	Chest compression	1	0	99	
147g.	Intubation	1	0	99	
147h.	Others (specify)……………………	1	0	99	
148.	Malformation	1	0	99	**If No or NR, go to 150**
149.	If Yes (multiple response)	Neural tube defects………………1<br>Cleft lip…………………………… 2<br>Cleft palate…………………………3<br>Club foot……………………………4<br>Hypospadias……………………… 5<br>Omphalocele……………..……… 6<br>Gastroschisis………………………7<br>Imperforate anus………………… 8<br>Other limb defects………………..9<br><br>Others (specify)…………………			
	Routine care of newborn	***Yes***	***No***	***NR***	
150.	Vitamin K	1	0	99	

151.	Body temperature	1	0	99	
152.	Respiratory rate	1	0	99	
153.	Medical examination of baby	1	0	99	
154.	Newborn transferred from labour room	Yes, SNCU/NICU………………......1<br>Yes, PNC…………………………..2<br>No………….………………………..0<br>Not recorded……..……………......99			**If SNCU/NICU, go to Part D**
155.	Outcome of the baby	Healthy……………………………….1<br>Improved…………………………....2<br>Referred to other facility………...3<br>**Died……..…………………………… 4**<br>Absconded……………………..………5<br>Stillbirth……………………………...7<br>DOPR……………………………...8			**If other than died goto q158**
156.	Date of death	_ _ _ _ _ _ _ _			
157.	Cause of death (multiple response)	Neonatal sepsis/infection………… 1<br>Meconium aspiration syndrome….2<br>Birth asphyxia……………………..3<br>Respiratory distress syndrome…..4<br>Hypoglycaemia………………… 5<br>Low birth weight (LBW)…………6<br>Preterm……………………………7<br>Congenital malformation………...8<br><br>Others (specify)…………………..			
158.	Date of discharge (AD) dd/mm/yyyy	_ _ _ _ _ _ _ _			

Confidential: For Review Only

Confidential: For Review Only
